# „Zum Verrücktwerden“. Die Generierung feministischer Psychiatriekritik am Beispiel der Zeitschrift *Courage*, 1978–1980

**DOI:** 10.1007/s00048-024-00405-1

**Published:** 2024-11-14

**Authors:** Susanne Doetz

**Affiliations:** Institut für Geschichte der Medizin und Ethik in der Medizin, Charité-Universitätsmedizin Berlin, Berlin, Deutschland

**Keywords:** Courage, Psychiatriekritik, Wissensgenerierung, Psychiatrieerfahrung, Frauenbewegung, Courage, Criticism of psychiatry, Knowledge generation, Patients’ experience, Women’s movement

## Abstract

Am Beispiel der feministischen Zeitschrift *Courage* zeigt der Beitrag, wie ihr partizipativer Herstellungsprozess eine psy-feministische Wissensgenerierung ermöglichte, die auch Frauen mit Psychiatrieerfahrung einschloss. Deren Beobachtungen, Wahrnehmungen und Deutungen verbanden die Zeitschriftenmacherinnen mit visuellen Darstellungen und einem Literaturkanon, der weit über das Feld der Psychiatrie(-kritik) hinausreichte. Anstelle medizinischer Psychopathologien implementierten die *Courage*-Frauen Schreibstile und Bildsprachen, die das subjektive Erleben von psychischen Leiden und psychischen Alteritäten in den Vordergrund stellten und es zur gesellschaftlichen Stellung von Frauen in Beziehung setzten. Gleichzeitig kamen in der Zeitschrift auch die Anbieterinnen feministischer Behandlungsmöglichkeiten zu Wort. Die Psychiatriekritik der *Courage* – so die These – zeichnete sich durch einen multiperspektivischen Ansatz aus, der das Thema „Frauen in der Psychiatrie“ aus dem engen Feld der Psychiatrie, Psychologie und Psychotherapie herauslöste und um künstlerische, patientinnen- und erfahrungsorientierte sowie gesellschaftskritische Perspektiven erweiterte.

## Abstract (en)

Using the example of the feminist magazine *Courage*, the article shows how its participatory production process enabled a psy-feminist knowledge generation that also included women with psychiatric experience. The magazine makers combined the women’s observations, perceptions and interpretations with visual representations and a canon of literature that extended far beyond the field of psychiatry (criticism). Instead of medical psychopathologies, the women of *Courage* implemented writing styles and visual languages, which emphasised the experience of mental suffering and alterity and related it to the social position of women. At the same time, *Courage* also presented alternative feminist treatments and therapies. The *Courage*’s critique of psychiatry was characterised by a multi-perspective approach that removed the topic of “women in psychiatry” from the narrow field of psychiatry, psychology and psychotherapy and expanded it to include artistic, patient- and experience-oriented and socio-critical perspectives.

## Psychiatrieerfahrene Frauen kommen zu Wort

Im April 1980, fünf Jahre nach dem Abschlussbericht der Psychiatrie-Enquete und ein halbes Jahr bevor mehr als 4.000 Menschen in Bonn für die Auflösung der Großkrankenhäuser demonstrierten (Reumschüssel-Wienert 2021: 148), gab die Frauenverlagsgesellschaft *Courage *das Sonderheft „Zum Verrücktwerden. Frauen in der Psychiatrie“ heraus. Damit reihte sie sich in jene Medienberichterstattung ein, die seit Ende der 1960er Jahre die Unterbringungs- und Versorgungssituation psychiatrischer Patient*innen in bundesrepublikanischen Anstalten skandalisiert hatte (Beyer 2022). Der Titel des Sonderheftes lässt sich zudem als Reminiszenz an psychiatriekritische feministische Werke lesen, wie das Buch *Frauen – das verrückte Geschlecht?* der US-amerikanischen Psychologin Phyllis Chesler (1974) oder das bundesrepublikanische Pendant *Wie Frauen „verrückt“ gemacht werden*, die überarbeitete Diplomarbeit der Psychologin Roswitha Burgard (1977). In beiden Büchern stellen die Autorinnen einen Zusammenhang zwischen der untergeordneten gesellschaftlichen Stellung von Frauen und ihrem psychischen Leiden sowie ihrer Psychiatrisierung her. Dennoch stellte das 84-seitige Sonderheft eine Besonderheit dar, denn ein großer Teil der darin abgedruckten Beiträge stammte von Frauen^1^ mit psychischen Leiden oder Krisen, die meisten von ihnen mit Psychiatrieerfahrung.^2^ Zwar hatten feministische Zeitschriften bereits zuvor (und auch danach) die Werke psychiatrie- oder krisenerfahrener Autorinnen wie Sylvia Plath, Unica Zürn oder Charlotte Perkins Gilman vorgestellt oder in Auszügen veröffentlicht.^3^ Auch fand eine breit gefächerte Auseinandersetzung mit der Psychoanalyse und mit einigen der von ihr verwendeten Konzepte statt.^4^ Die „Figur der verrückten Frau“ hatte, wie Annette Schlichter (2000: 20) aufgezeigt hat, eine „konstitutive Funktion“ für die feministische Theoriebildung. Dass allerdings psychiatrieerfahrene Frauen in einer Zeitschrift in diesem Umfang und ohne die Vermittlung einer Rezensentin oder Interviewerin zu Wort oder bildlichem Ausdruck kamen, war ein Novum.^5^ Gleichzeitig beschränkte sich die *Courage* in diesem Sonderheft nicht auf Erfahrungsberichte aus der Psychiatrie, sondern bot neben einer vielseitigen künstlerischen Auseinandersetzung mit der Thematik auch der Selbstdarstellung feministischer Therapie‑, Beratungs- und Selbsthilfeangebote Raum und verwies auf einen breiten Psy^6^-Literaturkanon. Mit diesem multiperspektivischen Ansatz lösten die *Courage*-Frauen^7^ das Thema aus den engen Grenzen der klassischen P‑Disziplinen Psychiatrie, Psychologie und Psychotherapie. Gleichzeitig vollzog die Zeitschrift den Spagat, betroffenen Frauen eine Stimme zu geben und diese Stimmen gleichzeitig in den Kontext einer feministischen Gesellschafts- und Psychiatriekritik zu stellen.

In diesem Artikel möchte ich am Beispiel der *Courage*, die ihre Leserinnen auf vielfältige Weise in den Herstellungsprozess der Zeitschrift einband, untersuchen, wie Frauen – in Anlehnung an Kathy Davis (2007) – zu *epistemic agents* wurden. Darunter versteht Davis, dass Frauen in der Lage sind, Wissen über sich selbst und ihre Umwelt zu erwerben, zu interpretieren, kritisch zu reflektieren und ihre Erfahrungen als Ressource für kritisches Wissen zu mobilisieren. Dies bedeutet weder, dass Davis diese Erfahrungen als letzte Instanz einer authentischen Wahrheit ansieht, noch, dass sie in Bezug auf die Agency der Frauen die Bedeutung struktureller Abhängigkeiten und diskursiver Einschreibungen leugnet (ebd.: 14, 135–139).^8^

Anne Kwaschik (2023) beschreibt die Frauenbewegung nicht nur als gesellschaftspolitisches, sondern auch als epistemisches Projekt, in dem weiblicher Erfahrung eine zentrale Bedeutung zukam. Dieses beinhalte verschiedene kollektive Praktiken wie gemeinsames Schreiben, Selbstuntersuchungen sowie das Sammeln von Daten. Die hieraus generierten Erfahrungen seien wiederum in die international zirkulierende feministische Literatur eingeflossen und dort reflektiert worden. Dies sei zugleich ein eminent politisches Unterfangen gewesen, stellte es doch – dem eigenen Verständnis nach – zentrale wissenschaftliche Kategorien wie Neutralität und Objektivität infrage und betonte die Situiertheit von Wissen.

Anhand der kollektiven und partizipativen Praxis des Zeitschriftenmachens möchte ich zeigen, wie ein solches Gegenwissen^9^ hervorgebracht und gleichzeitig aktivistisch eingesetzt wurde.^10^ Die Zeitschriften boten einen Raum der wechselseitigen Bezugnahme, griffen aber auch Impulse von „außen“ auf und wirkten über die Frauenbewegung hinaus. Sie waren nicht nur Mittel der Informationsvermittlung, vielmehr ging ihre Produktion mit sozialen Interaktionen und Praktiken einher. So war, um dem Paradigma des feministischen Erfahrungswissens gerecht zu werden, ein enger Austausch zwischen Zeitschriftenmacherinnen und Leserinnen notwendig. Dieser äußerte sich nicht nur in den abgedruckten Beiträgen der Leserinnen, sondern auch in detaillierten Auskünften der Macherinnen über das Innenleben der Zeitschrift.^11^

Als Quellen dienen mir neben feministischen Zeitschriften aus den 1970er und 1980er Jahren Archivalien aus dem Berliner Frauenforschungs-, -bildungs- und -informationszentrum (FFBIZ), dem Hamburger Institut für Sozialforschung (HIS) und dem Bundesarchiv in Koblenz.^12^

Zunächst beschreibe ich anhand der feministischen Zeitschriftenproduktion der 1970er und 1980er Jahre verschiedene Praktiken und Faktoren, die eine feministisch-erfahrungsbasierte Wissensgenerierung ermöglichten. Mit welchen Mitteln dies in der *Courage* umgesetzt wurde, untersuche ich anschließend am Beispiel einer vierteiligen Serie der Zeitschrift über Psychiatrie und Anti-Psychiatrie sowie anhand des erwähnten Sonderheftes. Abschließend diskutiere ich die daraus hervorgehenden Schnittmengen von Frauenbewegung und Psychiatriekritik.

## Feministische Zeitschriftenproduktion

Dem bedruckten Papier, sei es in Form einer Zeitschrift, Broschüre oder in Form eines Buches, kam in der autonomen Frauenbewegung der 1970er und 1980er Jahre eine zentrale Bedeutung zu. Das heißt nicht, dass Videos, Schallplatten, Audiokassetten oder Filme belanglos waren,^13^ aber sie wirkten nicht auf dieselbe Weise struktur- und netzwerkbildend. Denn die Produktion und Verteilung von Zeitschriften, Broschüren und Büchern ging mit der Gründung von Redaktionskollektiven, feministischen Nachrichtendiensten, Vertrieben und Verlagen sowie Frauendruckereien und Frauenbuchläden einher. Diese förderten die Netzwerkbildung, wirkten identitätsstiftend und dienten teilweise auch als Einnahmequelle. Die meisten dieser Kollektive und Projekte waren Teil einer alternativen Ökonomie, die sich in den 1970er Jahren herausbildete. Ihre Arbeit war dem Anspruch nach hierarchiefrei, gebrauchswertorientiert und verfolgte politische Ziele. Daher standen die Akteurinnen gegenüber dem eigenen Milieu auch immer unter Rechtfertigungsdruck.^14^

Bereits in der Frühphase der autonomen Frauenbewegung – zwischen 1970 und 1976 – erschienen im deutschsprachigen Raum etwa 50 feministische Zeitschriften (Schallner 2022).^15^ Claudia Weinel listet in ihrer Magisterarbeit für den Zeitraum von 1970 bis 1984 insgesamt 132 feministische Zeitschriften in der Bundesrepublik auf. Mit einer Ausnahme durften für diese nur Frauen Beiträge verfassen.^16^ Ein Maximum war 1983 erreicht; in diesem Jahr erschienen 59 verschiedene Publikationen (Weinel 1984: 46–49, 105–122).^17^ Obwohl einige Zeitschriften – über den gesamten Zeitraum betrachtet – nur eine kurze Lebensdauer hatten, zeigen diese Zahlen doch, dass die Herstellung eines solchen Produkts damals eine verbreitete Praxis war. Dafür spricht auch, dass die Volkshochschule Düsseldorf einen Kurs mit dem Titel „Wie mache ich eine Frauenzeitung?“ anbot.^18^

Neben fachspezifischen Zeitschriften wie der bereits 1974 gegründeten *Frauen und Film*, der Literaturzeitschrift *Mamas Pfirsiche* oder der vom feministischen Gesundheitszentrum in West-Berlin herausgegebenen Zeitschrift der Selbsthilfebewegung *Clio* gab es auch überregionale Zeitschriften ohne Fachspezifik wie die *Courage, Die Schwarze Botin* oder die *Emma*. Vor allem aber existierte eine Vielzahl regionaler Periodika, die sich keinesfalls nur auf die feministischen Zentren in West-Berlin, München, Frankfurt am Main, Köln, Hamburg und Bonn beschränkten, sondern auch in anderen – vorwiegend universitären – Orten erschienen. Zu nennen sind hier beispielsweise die *Aachener Frauenzeitung*, die *Donna Klara* aus Darmstadt oder *Hexengewitter* aus Emden. Darüber hinaus gab es Zeitschriften, die sich speziell an lesbische Frauen richteten, wie die *Partnerin, Unsere kleine Zeitung* und der *Lesbenstich*, oder deren Macherinnen sich als „Frauen“ bezeichneten, die „mit Männern schlafen und es immer noch nicht aufgegeben haben“ (Anagan 1984: 3).^19^

Zur Konkurrenz und zu den inhaltlichen Auseinandersetzungen – insbesondere zwischen der *Courage*, die sich als Sprachrohr der autonomen Frauenbewegung verstand, der *Emma*, die sich an eine breite Öffentlichkeit richtet, sowie der *Schwarzen Botin*, die den Anspruch kritischer Konfrontation vertrat – ist bereits publiziert worden (Deininger 2004; Vukadinović 2021). Katharina Lux (2022) hat zudem die Bedeutung dieser Debatten für die feministische Theoriebildung herausgearbeitet. Ich möchte im Folgenden einen anderen Fokus wählen, indem ich verschiedene Praktiken rund um die Produktion der Periodika in den Blick nehme: Zum partizipativen Anspruch des Zeitungsmachens, den viele feministische Redaktionen teilten, gehörte es, möglichst viele Frauen zum Schreiben zu ermutigen. Dies führte dazu, dass Redaktionskollektive Auswahlkriterien für die ihnen zugesandten Manuskripte entwickeln mussten. Darüber hinaus entstand eine Diskussionskultur, die immer wieder das Spannungsfeld zwischen Frauensolidarität und feministischer Kritik auszuloten hatte. Außerdem war es notwendig, ökonomische Logiken mit politischen Ansprüchen in Einklang zu bringen. Trotz aller Abgrenzungsdebatten und Boykottaufrufe teilten Zeitschriftenmacherinnen im Rahmen von Zeitungstreffen Informationen miteinander, tauschten Artikel aus, warben füreinander und vernetzten sich.^20^

## „Schreib das auf, Frau“

*„Schreib das auf, Frau“*^21^* – Die Überwindung der Sprachlosigkeit* hieß eine von Gabriele Dietze herausgegebene Anthologie, die 1979 im Hermann Luchterhand Verlag erschien. Das Buch sollte ein autonomes weibliches Sprechen ermöglichen, dem nicht die Vorstellung eines naturalisierten, essenzialistischen Konzepts von Weiblichkeit zugrunde lag, wohl aber das Bewusstsein einer Differenz der Geschlechter in einer patriarchalen Ordnung (Dietze 1979). In der autonomen Frauenbewegung existierten verschiedene Strategien zur Überwindung der wiederholt beklagten Sprachlosigkeit: In München, West-Berlin, Köln und Bremen fanden bundesweite Treffen schreibender Frauen statt, auf denen die Teilnehmerinnen sowohl eigene Texte vorstellten und gemeinsam diskutierten als auch über „weibliche Sprache“, die gesellschaftliche Situation schreibender Frauen, Qualitäts- und Publikationskriterien und die literarische Tradition von Frauen debattierten. Die Bandbreite der Themen, das Changieren zwischen fiktivem Schreiben und Schreiben als Selbsterfahrung, die unterschiedlichen Standpunkte sowie die häufig lose Organisationsform boten dabei durchaus Konfliktpotenzial.^22^ Auch auf lokaler Ebene gründeten sich Arbeitsgruppen schreibender Frauen. Aus einer dieser Arbeitsgruppen heraus entstand an der Volkshochschule Dortmund der Kurs „Schreiben befreit“. Die beiden feministischen Kursleiterinnen verfolgten ein pädagogisches Konzept und arbeiteten methodisch mit assoziativem und auch spontanem Schreiben. Die Intensität der Textkritik erfolgte in Abstimmung mit den jeweiligen Autorinnen. Die Grenze zwischen „literarischem“ und „therapeutischem“ Schreiben seien in diesen Kursen – so die Kursleiterin Gisela Schalk (1988) – fließend. Nichtsdestotrotz blieb die Art und Weise, wie Schreiben verstanden werden sollte – ob als Ausdruck von Selbsterfahrung, künstlerischer Schaffensprozess oder wissenschaftliche Reflexion – ein wichtiges Distinktionsmerkmal innerhalb der Frauenbewegung und Gegenstand der Auseinandersetzung (siehe u. a. Strobl 1988).

Hinsichtlich der Beteiligung ihrer schreibenden Leserinnen verfolgten die einzelnen Zeitschriften unterschiedliche Konzepte. Während die Redaktion der *protokolle. informationsdienst für frauen* zunächst jeden zugesandten Artikel abdruckte, sofern er nicht veraltet war, und ihre Aufgabe hauptsächlich im Tippen der Texte und im Zusammenstellen der Hefte lag (Anonym 1977), stellten die Herausgeberinnen der *Schwarzen Botin* klar, dass sie nur abdruckten, was sie selbst interessierte ([Goettle] 1976). Die *Courage*-Redaktion wiederum informierte ihre Leserinnen haarklein, wie sie mit eingesandten Texten umging, beginnend mit der Bekanntgabe der Texteingänge über die Registratur und die zwischenzeitliche Ablage bis hin zur Verteilung auf die einzelnen Redaktionsfrauen. Die Texte wurden dann in der gemeinsamen Redaktionssitzung laut gelesen und diskutiert. Damit verbanden die *Courage*-Frauen folgende Intention: „Das Laut-Lesen ist uns wichtig, weil so die Texte über das Zuhören und Mitlesen noch eingänglicher werden, und die Kommunikation zwischen uns noch spontaner und direkter ist. Jede hört anders, hakt woanders ein“ (Klarner 1980: 2). Anschließend konnten Änderungen und Kürzungen vorgeschlagen werden, die die zuständige Redaktionsfrau dann der Verfasserin kommunizierte. Befand die Redaktion einen Artikel für „zu ungenau, langweilig, sprachlich oder inhaltlich so trocken“ (ebd.), lehnte sie ihn ab und schickte ihn der Autorin mit der entsprechenden Begründung zurück.

Das Vorlesen war somit ein kollektives und kommunikatives Prozedere, das einen Rückgriff auf die vielfältigen Erfahrungen der Teilnehmerinnen ermöglichte. Allerdings konnte es angesichts bestehender informeller Hierarchien – so Birgit Klarner, als sie nach zweieinhalb Jahren frustriert die *Courage*-Redaktion verließ – auch die „totale Kontrolle“ (Klarner 1982: 56) bedeuten, da eine einzelne Frau nicht selbstständig über einen Text urteilte und entschied, sondern ihre Verantwortlichkeit an die dominierenden Frauen im Kollektiv abgab. Gerade dieses „Alleszusammenlesen“ (ebd.) am Redaktionstisch habe keinen Raum für Auseinandersetzungen und neue Ideen gelassen, so Klarner. Dass solche Interna – im Herbst 1981 verließen neben Klarner noch fünf weitere Frauen die *Courage *– wiederum detailliert in der Zeitschrift abgedruckt und von verbleibenden *Courage*-Frauen kommentiert wurden (Dormagen et al. 1982), lässt sich auf den eigenen Anspruch auf Transparenz zurückführen: Die Leserinnen sollten am Innenleben der Zeitschrift teilhaben können. Es spricht aber auch für einen hohen Rechtfertigungsdruck gegenüber dem eigenen Milieu.

Der Ablauf der gemeinschaftlichen Sitzungen legt zudem nahe, dass die *Courage*-Redaktion eine eher lenkende als schreibende Funktion hatte. Immerhin stammten etwa zwei Drittel aller Beiträge von den Leserinnen^23^ der Zeitschrift. Diese konnten außerdem an den einmal im Monat – jeweils nach Erscheinen einer Ausgabe – stattfindenden öffentlichen Redaktionssitzungen teilnehmen und dort ihre Kritik äußern (Schneegass 1993).

## Layout und Finanzen

Frauen kamen in den feministischen Zeitschriften nicht nur zu Wort, sondern sandten auch Bilder ein. Die Zeitschriftenmacherinnen griffen zudem auf bereits bekannte visuelle Gestaltungen oder auf in anderen Kontexten bereits verwendete Abbildungen zurück. Den sich Wort für Wort aus den Texten entwickelnden Narrativen stellten sie somit unmittelbar wirkende Fotos, Zeichnungen oder andere visuelle Formate zur Seite. Dabei handelte es sich nicht um simple Illustrationen, vielmehr konnten sie als Interpretationen oder Weiterentwicklungen der Texte oder gar als eigenständige und mehrdeutige Geschichten gelesen werden. Damit hatte das Layout nicht nur eine gestaltende, sondern auch eine inhaltliche Funktion.

Die seit den 1960er Jahren vereinfachten und verbilligten Möglichkeiten zur Herstellung und Reproduktion von Druckerzeugnissen waren eine wichtige Voraussetzung für die Entwicklung einer links-alternativen und feministischen Gegenöffentlichkeit (Stadler et al. 2020).^24^ Im Hinblick auf die hier untersuchten feministischen Zeitschriften bestand häufig eine dergestaltige Arbeitsteilung, dass der Druck bei feministischen oder linken Druckereien erfolgte, die dann wiederum in den Zeitschriften beworben wurden.^25^ Das Layout übernahmen hingegen zu großen Teilen die Zeitschriftenmacherinnen selbst. Es wurde meist in kollektiver Handarbeit erzeugt. Für die monatlich und in den ersten Jahren zum Preis von drei DM erscheinende *Courage* gibt Gisela Notz (2007: 43f.) an, dass „Schere, Fixo-Gum und Letra-Set“ sowie (angesichts der wiederkehrenden Auseinandersetzungen mit den Redakteurinnen) Durchsetzungsvermögen zu den wichtigsten Produktionsmitteln der Layouterinnen zählten.^26^ Diese relativ einfach zu handhabenden Hilfsmittel ermöglichten die Zeitschriftenproduktion an einem schlichten Tisch,^27^ wobei die *Courage *sich von dem in den frühen Jahren der feministischen Presse üblichen Flattersatz wegbewegte und stattdessen einen Composer nutzte – das heißt eine Schreibsetzmaschine, mit der Wortabstände angepasst und Texte im Blocksatz wiedergegeben werden konnten. Damit professionalisierte die *Courage *nicht nur ihr Erscheinungsbild, sondern trug auch zu einer Ästhetisierung feministischer Inhalte bei.

Die technischen Hilfsmittel können ebenso wie die partizipative Arbeitsweise nicht losgelöst von ökonomischen Faktoren betrachtet werden. In den meisten Fällen war das Erstellen der feministischen Zeitschriften eine weitgehend unbezahlte Tätigkeit. Aufwendige eigene Recherchen konnten die Macherinnen nicht unternehmen, sodass sie auf die Beiträge ihrer Leserinnen angewiesen waren.

Im Vorfeld des Frauenzeitungstreffens in West-Berlin 1988 hatten die Organisatorinnen eine Umfrage ausgewertet, an der 23 deutschsprachige Zeitschriften (22 aus der Bundesrepublik und eine aus der Schweiz) teilgenommen hatten. Eines der Ergebnisse war, dass es nur bei fünf dieser Zeitschriften jeweils eine bezahlte Stelle gab. Nur in zwei Fällen konnte diese von der jeweiligen Zeitung selbst finanziert werden, in einem Fall bezahlte die Stelle zu zwei Dritteln das Arbeitsamt, in einem weiteren handelte es sich um eine staatlich finanzierte Arbeitsbeschaffungsmaßnahme und in einem Fall war der Zeitung die Stelle „geschenkt“^28^ worden. Angesichts des hohen Maßes an unentgeltlicher Arbeit, die hier geleistet wurde, verwundert es nicht, dass die Zeitungsmacherinnen in ihrer Mehrzahl Studentinnen oder erwerbslose Frauen waren.^29^ Die Macherinnen der 1976 gegründeten *Courage*, die 1984 Konkurs anmelden musste, experimentierten dagegen nach mehr als einem Jahr ohne Bezahlung mit verschiedenen Verdienstmodellen. Im April 1978 erhielt jede *Courage*-Frau pro Viertelstelle 200 D‑Mark, zuzüglich ihrer jeweiligen Kosten für Miete, Strom und Telefon sowie abzüglich etwaiger Verdienste aus anderer Arbeit. Autorinnen und Fotografinnen konnte die Zeitschrift teilweise die Auslagen erstatten (Anonym 1978a; Schneegass 1993). Dennoch waren auch in der *Courage* professionelle Journalistinnen und Grafikerinnen in der Minderheit. Im Nachhinein entpuppte sich die Zeitschrift allerdings als „Schreibeschule für Autorinnen“, wie es die *Courage*-Mitbegründerin Sibylle Plogstedt einmal formulierte (zit. n. Schneegass 1993: 81). Die Zeitschrift bot aber auch eine Verdienstmöglichkeit in Zeiten befürchteter oder – wie im Fall Plogstedts – tatsächlicher Berufsverbote aufgrund des sogenannten Radikalenerlasses (Plogstedt 2006: 33).^30^ Die Ökonomie in Form von Abonnements, Anleihen und Spenden war darüber hinaus ein weiteres Bindeglied zwischen Macherinnen und Leserinnen der *Courage *(Anonym 1982b).

## Generierung von Psy-Wissen in der Courage

Der im vorherigen Abschnitt beschriebene partizipative Ansatz der *Courage *war auch für ihre Darstellung von Psychiatrie- und Psychotherapiekritik, von psychischen Leiden sowie feministischen Behandlungsmethoden von entscheidender Bedeutung. Schneegass (1993: 81) zufolge sei der Anlass für das 1980 von der *Courage* herausgebrachte Sonderheft über „Frauen in der Psychiatrie“ eine überwältigende Flut von Briefen und eingesandten Berichten zu diesem Thema gewesen. Es ist anzunehmen, dass diese wiederum eine Reaktion auf die vier bereits 1978 publizierten Schwerpunkthefte über (Anti‑)Psychiatrie waren.^31^ Zu diesem Zeitpunkt hatte die *Courage *eine Auflage von circa 70.000 Exemplaren. Sie wurde neben Abonnements und Handverkauf vor allem über den Zeitungshandel vertrieben und war somit nicht nur in den einschlägigen Frauenbuchläden, sondern auch an Zeitungskiosken erhältlich (Notz 2007: 28; Schneegass 1993: 84).

Von den *Courage*-Frauen, die das Sonderheft gestalteten und bearbeiteten, hatte einzig Christa Müller als Psychologin und Mitbegründerin der Psychosozialen Initiative für Frauen (PSIFF) einen direkten beruflichen Bezug zum Thema.^32^ Bis auf wenige Ausnahmen – zwei kritische Artikel von Plogstedt über den Einsatz von Elektroschocks als therapeutische Methode, einen Beitrag über das PSIFF, an dem Müller offensichtlich mitgearbeitet hatte, und ein Interview zweier *Courage*-Frauen mit den Frauen des Projekts „Therapie und Beratung für Frauen“ (TUBFF) – stammten die Texte durchweg nicht von der Redaktion. Dies stand im Einklang mit der Position der *Courage*, betroffene Frauen selbst zu Wort kommen zu lassen und deren Erfahrungen an erste Stelle zu setzen. Dahinter mochte die Vorstellung gestanden haben, dass nur sie in der Lage seien, „authentisch“ zu sprechen.^33^ Die Vorgehensweise kann aber auch als bewusster politischer Akt der Selbstvertretung gedeutet werden, der sich gegen bestehende Machtverhältnisse richtete. Dafür spricht folgende in der Nullnummer der Zeitung formulierte Zielsetzung: „Frauen sollen durch COURAGE Anregungen bekommen, sich mit ihrer und der Situation anderer Frauen auseinanderzusetzen, sollen ihre eigenen Veränderungen sehen und beschreiben, mehr Frauen dazu ermutigen, für ihre Interessen einzutreten.“ (*Courage*-Redaktion 1976: 2).

Bei dem von der *Courage* praktizierten partizipativen Verfahren lässt sich nicht völlig ausschließen, dass sich unter den Schilderungen in erster Person auch Fiktionen befinden.^34^ Es gibt auch keine Unterlagen, anhand derer sich die Auswahl der Texte für die hier besprochenen Hefte oder deren weitere Bearbeitung durch die Redaktion nachvollziehen lässt. Es geht hier also darum, herauszuarbeiten, wie die Macherinnen der jeweiligen Ausgaben mit dem von ihnen verwendeten Material ein dezidiert psychiatriekritisches feministisches Wissen kuratierten und somit generierten und wie sie diesen Prozess der Wissensgenerierung gleichzeitig zu einem empowernden und politischen Akt machten. Sie kritisierten dadurch nicht nur die herkömmliche Psychiatrie und deren Behandlungsmethoden – die Hefte sind vielmehr als Versuch anzusehen, einen (anderen) Umgang mit psychischem Leiden oder psychischen Alteritäten zu finden. Dazu zählt auch, dass die *Courage* mit den hier versammelten Beiträgen Sprachstile implementierte und Bilder generierte, mit denen über diese Erfahrungen gesprochen und diese repräsentiert werden konnten.

Die Annäherung an das Thema (Anti‑)Psychiatrie und Therapie erfolgte über verschiedene Textgattungen, die durch Fotos, Zeichnungen und den Abdruck von Gemälden ergänzt wurden: Neben Erfahrungsberichten in der ersten Person, Tagebucheinträgen, Briefen und Selbstdarstellungen von Gruppen enthielten die Hefte auch Interviews, Gedichte und Erzählungen sowie einige wenige theoretische Beiträge.

## Erfahrungsberichte, Selbstdarstellungen und Interviews

Die Schilderungen der psychiatrie- und psychotherapieerfahrenen Frauen waren zum Teil mit vollem Namen gezeichnet, zum Teil nur mit Vornamen, Initialen oder mit einer Personenbeschreibung wie „eine 18-jährige Schülerin“ (1980: 27). Diese Namen wurden auch im Impressum unter „Autorinnen“ beziehungsweise „Mitarb. d. Nr.“ aufgeführt. Ob es sich bei den vollständigen Namen um Klarnamen oder Pseudonyme handelt, wurde nicht ausgeführt. Im Impressum wurden die Namen aller Beiträgerinnen alphabetisch aufgelistet – unabhängig davon, ob es sich um Patientinnen, in der Psychiatrie Beschäftigte oder Therapeutinnen handelte. Alle standen gleichberechtigt in einer Reihe, womit die Redaktion einen Kontrapunkt zur hierarchischen Organisation der psychiatrischen Versorgungslandschaft setzte.

Ein Großteil der Beiträge gibt einen Einblick in Tagesabläufe und Routinen in der Psychiatrie aus der Perspektive der Insassinnen. Insgesamt zeichnen sie ein eher düsteres Bild der erwähnten Anstalten: Vom „unverblümte[n] Horror der Püschiatrie“ (Stepken 1980: 11) ist die Rede, von „Arroganz und Besserwisserei“ (Rebecca 1980: 16) der behandelnden Ärzt*innen, von der Identität, die „bei der Einweisungsprozedur“ „an der Pforte“ abgegeben werden muss (K. 1980), von physischer Gewalt durch das Personal (Rebecca 1980; Anonym 1980a; E. 1980; Cornelia 1980), von starken Nebenwirkungen der Medikation (Stepken 1980; Rebecca 1980; Susy 1980), von homophoben Bemerkungen einer Sozialarbeiterin (Discher 1980) und vom Ekel vor Mitpatient*innen (Anonym 1980a). Die Autorin „Rebecca“ bringt es auf den Punkt, wenn sie direkt zu Beginn ihres Beitrags schreibt: „Im Frühjahr letzten Jahres bin ich aufgrund einer schweren psychischen Krise, die auf einige Monate Drogenabhängigkeit folgte, freiwillig in eine psychiatrische Klinik gegangen. Diesen Entschluß habe ich in den 105 Tagen, das sind 2520 Stunden und über 15000[0] Minuten, bereut“ (Rebecca 1980: 14).

Immer wieder beschreiben die Frauen die erlebte Ohnmacht, womit die Berichte weitgehend das damals aufkommende Bild einer „totalen Institution“ (Goffman 1973) bestätigen.^35^ Auch visuell wird dies beispielsweise in einer Grafik von Katarina Albinghausen ausgedrückt, die eine junge, in Weiß gezeichnete Frau vor schwarzem Hintergrund zeigt, die aus einer Anstalt wegläuft, aus der eine weiße Hand nach ihrem Bein greift (*Courage *Sonderheft 1980: 12).

Allerdings finden sich vereinzelt auch andere Darstellungen. So schildert „eine 18-jährige Schülerin“ (1980: 27) in ihren tagebuchartigen Aufzeichnungen zunächst, wie ausgeliefert sie sich im Krankenhaus fühlt und wie ihre Selbstbestimmung „mit Füßen getreten“ wird. Am Ende jedoch hat sich die Klinik für sie in ein Heim gewandelt – „eine feste Wand zum Anlehnen oder Dagegenrennen, sie und der Psychiater geben nicht nach und halten mich. Vielleicht hat mir das auch mal gefehlt, etwas oder jemand, wo ich nicht durchkam“ (ebd.).

Der letzte Satz in diesem Zitat belegt außerdem, dass sich die eingesandten Texte nicht auf Beobachtungen oder die Beschreibung von Gefühlen und Wahrnehmungen beschränken, sondern die Verfasserinnen all dies auch deuten. Dabei greifen sie unter anderem auf damals gängige Erklärungen aus Psychologie und Medizin zurück. So verweist die Schülerin auf „so ein Nähe-Distanz-Problem“ (ebd.). Auch nutzen manche Frauen medizinische Diagnosen, um ihre Leidenszustände zu erklären. Das folgende Zitat zeigt, wie eine Frau die damals übliche Unterscheidung zwischen einer reaktiven und einer endogenen Depression zur Krankheitsdeutung heranzog und für sich interpretierte: „Bei allen Gefühlen steht überhaupt Angst an erster Stelle. Diese Angst war bei mir eigentlich nur von Außen beeinflußt. Alles, was ich so erlebt habe, hat sie hervorgerufen. Es gibt nämlich auch Depressionen von innen. Diese sind schwerer heilbar. Während eine andere Umgebung mich oft gesund gemacht hat“ (Petersen 1978: 10). Eine weitere Frau beschreibt die „Hölle“ ihrer „neurotischen Depression“, in der Tabletten die einzige Stütze seien (Katharina 1980: 60).

Es finden sich auch Beispiele von Frauen, die psychiatrische Diagnosen zurückwiesen. So spricht Rebecca (1980: 16) von einer „sogenannten Psychose-Diagnose“, eine weitere Frau schreibt von ihrer „angeblich endogenen“, aus ihrer Sicht aber „iatrogenen Erkrankung“ (Stepken 1980: 13). Darüber hinaus setzen einige Frauen ihre Leiden auch in Beziehung zu ihren Lebensumständen, wie etwa die Ehe mit einem Alkoholiker (Stepken 1980), Beziehungs- und WG-Probleme verbunden mit regelmäßiger transzendentaler Meditation (Susy 1980), Misshandlungen in Kindheit und Jugend einschließlich einer Vergewaltigung (E. 1980: 61) oder ein genormtes Hausfrauenleben (Cibach 1980: 70). Damit identifizierten die Frauen Lebensumstände als krankmachend, wie sie zum Teil auch Roswitha Burgard (1977) in ihrer für die westdeutsche Frauenbewegung grundlegenden Studie *Wie Frauen „verrückt“ gemacht werden* beschrieb. Die erwähnte Schülerin nimmt sogar direkt auf dieses Buch Bezug, wenn sie resümiert: „Naja, Hauptsache, frau läßt sich nicht verrückt machen“ (Eine 18-jährige Schülerin 1980: 27).

Die Autorinnen der Beiträge geben nicht nur einen Einblick in ihre Verfassung und Erfahrungen, sondern beschreiben und deuten auch umfassend das Verhalten ihrer Mitpatient*innen. Diese Schilderungen sind geprägt von Mitleid, Interesse und Angst bis hin zu Ekel und Abwertungen gegenüber Langzeitpatientinnen. So schreibt eine Frau selbstkritisch:

Sie [manche der Langzeitpatientinnen] sind nur das niedrige Lebewesen, grausige Scherze der Natur, sinnloses Leben. Und genau so werden sie hier vom Personal aufgefaßt. Eine junge Schwesternschülerin lernt dies bald. Wie soll ich dies sich immer tiefer einnistende Gefühl der Minderwertigkeit in mir loswerden, wenn es ohnehin in mir drin ist? Es bleibt nur noch die Arroganz, das Bedürfnis der Geltung, der kleinen Geltung (Anonym 1980a: 34).

In einem anderen Beitrag wird nur durch einen einzigen Hinweis deutlich, dass die Schreiberin selbst Patientin auf einer psychiatrischen Station ist. Sie schreibt nicht über ihre eigenen Erfahrungen, sondern geht auf die Wahninhalte ihrer Mitpatientinnen ein. Diese interpretiert sie als eine andere Form von Wahrheit und nicht etwa als unverständlich (Anonym 1980b).

In der *Courage* kamen jedoch nicht nur Frauen zu Wort, die sich als Patientinnen in der Psychiatrie aufgehalten hatten, sondern auch Personen, die dort arbeiteten. Bereits in der Ausgabe vom April 1978 erschienen die Schilderungen einer Krankenschwester sowie einer Assistenzärztin, die sich beide in der Deutschen Gesellschaft für Soziale Psychiatrie engagierten und ihre Unzufriedenheit mit ihren Arbeitsverhältnissen in herkömmlichen Anstalten beschrieben. Insbesondere die Ärztin erschien hier eher als hilflose Helferin denn als Halbgott in Weiß (M. 1978: 43f.; S. 1978: 44–47).^36^ Dagegen wurden die verschiedenen feministischen therapeutischen Einrichtungen und Selbsthilfegruppen als positive Alternative dargestellt (Die TUBFF-Frauen 1980; PSIFF 1980; Blessing 1980).

Die hier geschilderten Erfahrungen ergaben somit kein abgewogenes Pro und Kontra Psychiatrie. Vielmehr ist ihr Abdruck als politische Intervention zu verstehen, als Skandalisierung der damaligen Anstaltspsychiatrie, die die *Courage *als Bestandteil patriarchaler Machtstrukturen verstand.

Darüber hinaus bleibt die Frage, welche Erfahrungen hier nicht repräsentiert wurden. So thematisierte beispielsweise keine der in Ich-Form schreibenden psychiatrie- und/oder therapieerfahrenen Frauen das Hören von Stimmen. Dies wird zwar einmal bei einer Mitpatientin angesprochen, taucht aber nicht als Selbstbeschreibung auf, was verschiedene Interpretationen zulässt: Es kann darauf hindeuten, dass Stimmenhörerinnen keine Beiträge einsandten oder darauf, dass deren Beiträge aussortiert worden waren oder aber, dass hier eine Form der Selbstzensur vorliegt. Folglich war entweder die Hürde der Teilnahme zu hoch oder dieser Ausdruck des „Verrücktseins“ zu tabuisiert oder/und passte nicht in das Konzept, das vermittelt werden sollte. Auch Frauen mit geistiger Behinderung, die in den 1970er und 1980er Jahren vielfach noch in Psychiatrien untergebracht waren, kamen nicht zur Sprache.^37^ Eine Frau, die sich selbst als „alte Psychiatriekranke“ bezeichnet (Heiliger 1980: 58), bringt ihre Enttäuschung über das Sonderheft in einem Leserinnenbrief zum Ausdruck:

Ihr laßt nur die Jugend zu Wort kommen und die Heilmethoden in den Selbsthilfegruppen. Was ist mit denen, die schon kaputt gemacht worden sind und alt sind? Aus eigener Erfahrung weiß ich, daß man mir darum nicht helfen kann, weil man glaubt, ich wäre zu alt und zu sehr in den Händen der Behörden. Andererseits verlangt man von mir Dinge, die ich verlernt habe, auf Anhieb zu bewältigen. Na ja, da läßt man die alte Tucke fallen (ebd.).

Die Benachteiligung von Personen mit chronischen psychischen Erkrankungen, die von den Neuerungen im psycho-sozialen Feld nicht oder nur wenig profitierten, ist auch ein Kritikpunkt, der in Bezug auf die Psychiatriereform der 1970er und 1980er Jahre geäußert wird (Reumschüssel-Wienert 2021:174).

## Visuelle Ausdrucksformen und Akzentuierungen durch Text-Bild-Anordnungen

**Abb. 1:**
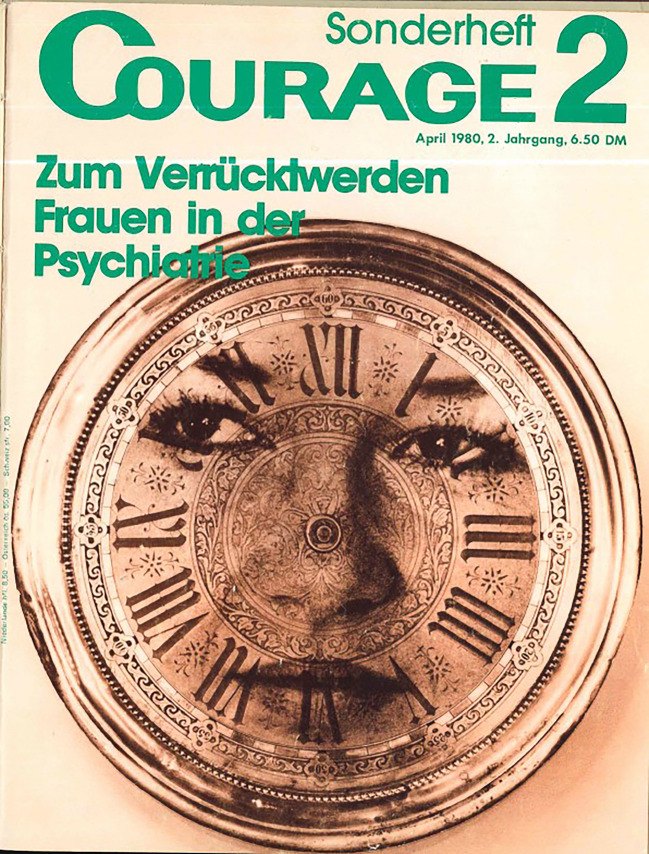
Das Titelblatt des *Courage Sonderheftes* 1980 unter Verwendung eines Bildes von Ingrid Pape (zu ihr konnten leider keine näheren Angaben gefunden werden). Bildnachweis: *Courage Sonderheft* 1980 2 (2)

Wie bereits erwähnt, wurden die Erfahrungen mit psychischem Leiden oder psychischer Alterität nicht nur in Form von Texten dargestellt, sondern auch visuell in Szene gesetzt. So zeigte das Titelbild des Sonderheftes (Abb. 1) ein mit dem fein ziselierten Ziffernblatt einer Uhr verschmolzenes junges Frauengesicht. Die Darstellung mutet surreal an und weckt zudem Assoziationen an Salvador Dalí. Damit verwies die *Courage* auf einen immer wieder hergestellten Zusammenhang zwischen Surrealismus und Wahnsinn. Ebenso wie surrealistische Künstler*innen Anleihen bei der Anstaltskunst nahmen, um hegemoniale Ordnungen generell infrage zu stellen, lässt sich auch dieses Titelbild nicht unbedingt als Verbildlichung einer inneren Krise lesen.^38^ Das Gesicht, das die Betrachter*innen direkt anschaut und ein leichtes Lächeln andeutet, lässt sich auch als Aneignung deuten; als Versuch, selbst über die eigene Zeit zu bestimmen und sich nicht einem von einer „patriarchalen“ Gesellschaft aufoktroyierten Zeitdiktat zu beugen. Auch die fehlenden Zeiger sprechen zum einen dafür, dass die Uhr ihrer Funktion, die Zeit anzugeben, nicht nachkommen kann und geben zum anderen dem Frauengesicht mit seinen kajalumrandeten Augen und geschminkten Lippen mehr Raum. Es sind hier jedoch auch andere Interpretationen vorstellbar. Diese Uneindeutigkeit offenbart meines Erachtens, dass das Titelblatt nicht bloß eine erläuternde Illustration war, sondern das Thema „Frauen in der Psychiatrie“ zwar aufgriff, es aber visuell weiterentwickelte. Gerade die Bilder in den hier besprochenen *Courage*-Heften vermochten die Thematik über das enge Feld einer Anstaltskritik hinaus zu erweitern und nahmen Frauen in ihrer Auseinandersetzung mit ihrer Umgebung und sich selbst in den Blick. In der Zeit eines vor allem männlich dominierten Kunstmarktes bot die *Courage *darüber hinaus Künstlerinnen – unabhängig davon, ob sie psychiatrieerfahren waren oder nicht – eine Publikationsmöglichkeit.^39^ So zeigte die *Courage *beispielsweise mehrere großflächige Grafiken Ingeborg Magieras mit ihren schemenhaften, teils archaisch anmutenden weißen Figuren auf schwarzem Hintergrund (*Courage Sonderheft* 1980: 48, 50f., 54–59).

Die Anordnung der Bilder gab den *Courage*-Frauen darüber hinaus die Möglichkeit, die Texte in einen feministischen und/oder psychiatriekritischen Kontext einzuordnen beziehungsweise solche Lesarten hervorzuheben. Dieser Effekt wurde teilweise durch eine entsprechende Überschrift noch verstärkt. So waren im Sonderheft von 1980 Sequenzen des Interviews von Inge Buck mit dem Zitat einer Stationsärztin betitelt: „Wir haben fast alles Hausfrauen“ (Buck 1980: 4f.). Diese Überschrift referierte auf den in feministischen Schriften hergestellten Zusammenhang zwischen dem Dasein als Hausfrau und psychischen Erkrankungen, der auch im Begriff „Hausfrauensyndrom“ (Burgard 1977) seinen Ausdruck fand und den Betty Friedan (1966) bereits in ihrem Bestseller *Der Weiblichkeitswahn *beschrieben hatte.^40^ Diesen Bezug nahmen zwei Bilder im Heft auf. Auf einer ganzseitigen Fotografie der Künstlerin Karen Plesur ist ein Wäscheschrank mit vier Fächern zu sehen. In dreien befinden sich mehr oder weniger zusammengeknüllte Kleidungsstücke, im zweiten Fach von unten ist eine nackte, zusammengekauerte Frau im Profil zu sehen, die ihr Gesicht verbirgt (*Courage Sonderheft *1980: 7). Eine nur undeutlich abgebildete Fotomontage der Videokünstlerin Sherrie Rabinowitz zeigt wiederum drei ältere nackte Frauen: Eine sitzt, die beiden anderen stehen hinter ihr, zwei von ihnen blicken direkt in die Kamera. Keine der drei Frauen ist vollständig abgebildet. Schräg über diesem Foto ist ein Bügeleisen montiert (ebd.: 10). Beiden Bildern fehlt jeglicher Bezug zur Psychiatrie, sodass sie für sich betrachtet auch unabhängig davon gelesen werden können – zum Beispiel im Falle von Rabinowitz als eine im Vergleich zu herkömmlichen Medien aufgrund des Alters der Modelle unübliche Repräsentation nackter Frauenkörper, die sich obendrein mit ihrem unerschrockenen, den Betrachter*innen zugewandten Blick nach damaliger Lesart dem Objektstatus entzogen.^41^

In dem von einer anonymen Autorin verfassten Artikel „Hier nimmt der Ekel alles Mitgefühl“ (Anonym 1980a) kamen zwei ikonische Fotos der Psychiatriekritik von Carla Cerati und Gianni Berengo Gardin zum Einsatz, die aus dem von Franco Basaglia und Franca Basaglia Ongaro (1969) herausgegebenen Fotoband *Morire di classe* stammen. Das eine Foto wurde in der psychiatrischen Klinik in Florenz aufgenommen und zeigt im Vordergrund links eine auf einer steinernen Bank ohne Lehne sitzende Frau in gestreifter Anstaltskleidung, wie sie mit leidendem Gesichtsausdruck die/den Fotografierende*n ansieht. Im Hintergrund sitzen vor einer Mauer mit bröckelndem Putz links eine Frau in Zwangsjacke auf einem Stuhl sowie rechts eine auf dem Boden kauernde Person (*Courage Sonderheft* 1980: 33; vgl. auch Foot 2015: 22).^42^ Auf dem anderen Foto ist am linken unteren Bildrand eine junge, schreiende, nach vorn gebeugte und dabei laufende Frau zu sehen, die sich auf den Bildrand zubewegt. Sie befindet sich auf dem leeren, parkähnlichen Innenhof der Anstalt in Gorizia mit ihren Bäumen und Bänken (*Courage Sonderheft* 1980: 34; Foot 2015: 27). Der Historiker John Foot wies darauf hin, dass *Morire di classe *als anti-institutioneller Fotoband konzipiert worden war, der explizit die Schrecken der Anstaltspsychiatrie aufzeigen sollte – sowohl in ihrer Architektur als auch in ihrer Einschreibung in die Körper ihrer Insass*innen. Dementsprechend wurden die zur selben Zeit stattfindenden Reformprozesse in Gorizia nicht ins Bild gesetzt. Foot (2015) problematisierte die Inszenierung der abgebildeten Anstaltsinsass*innen, die Gefahr laufe, die Abgebildeten zu Objekten zu machen und zu instrumentalisieren.^43^ Auch in der *Courage* erzeugte das Foto der drei in Florenz aufgenommenen Frauen durch die ins Bild montierte Überschrift sowie in Verbindung mit dem Text eine ambivalente Wirkung – schließlich konnte der thematisierte Ekel direkt auf die abgebildeten Frauen bezogen werden. Allerdings war auch eine andere Deutung möglich, wonach sich der Ekel auf die Anstalt bezog und eine entsolidarisierende Wirkung zur Folge habe und zu Vereinzelung führe, die sich im Bild durch die voneinander getrennten und sich nicht aufeinander beziehenden Frauen ausdrückt.

Wie für *Morire di classe*, so gilt auch für das *Courage*-Sonderheft, dass bei den Darstellungen psychiatrie- und therapieerfahrener Frauen düstere Abbildungen deutlich überwiegen: Leere Bänke versinnbildlichen Heimatlosigkeit, eine gefesselte Frau und Stacheldraht den Zwangscharakter der Institution und eine beschädigte weibliche Statue die Chancenlosigkeit. Besonders deutlich wird dies auch bei der Bildauswahl aus dem Fotoband der US-amerikanischen Fotografin Mary Ellen Mark und der Autorin und Sozialwissenschaftlerin Karen Folger Jacobs *Ward 81 *(1979), in dem Fotos aus der geschlossenen Frauenabteilung der Oregon State Mental Institution gezeigt werden.^44^ Mark und Folger Jacobs verbrachten 36 Tage auf der Station. In dieser Zeit interviewten und fotografierten sie die dort untergebrachten Frauen. Die in der Dokumentation abgedruckten Bilder zeichnen ein sehr heterogenes Bild dieser Station. Es werden nicht nur Fixierungen, der Umgang mit Elektroschocks und Zäune gezeigt, sondern auch lachende, tanzende und sich umarmende Frauen, ohne dass dahinter die Institution zum Verschwinden gebracht würde. Eine Frau, die auf einem Foto freundlich lächelt und auf dem anderen Grimassen schneidet, spielt offensichtlich mit dem Klischee von „Verrücktheit“. Diese Heterogenität und auch das stellenweise Spielerische bilden sich in der Auswahl der *Courage*-Frauen nicht ab. Sie übernahmen stattdessen elf Fotos, die einen eindeutig negativen Bezug hatten.^45^ Die überwiegend negativ konnotierte Bebilderung der Texte über Frauen in der Psychiatrie wird kontrastiert durch Abbildungen entschlossener, kämpferischer und einander zugewandter Frauen bei Artikeln, die von Widerstand gegen eine Therapie oder von feministischen Therapien oder Beratungen handeln. So ist neben Nadine Millers Abrechnung mit ihrem Therapeuten (1980) das Foto einer schwarz gekleideten Frau zu sehen, die mit großen Schritten entschlossen über einen Platz mit Kopfsteinpflaster schreitet. Der mit „Hier bin ich die Frau der Stunde“ überschriebene Text der West-Berliner Therapie- und Beratungsstelle für Frauen zeigt eine Frau in Kampfposition, und das Gespräch mit einer Frau aus einer PSIFF-Gruppe ist bebildert mit einem Foto der italienischen Künstlerin Marcella Compagnano. Es zeigt die Köpfe zweier lachender junger Frauen, von denen die eine ihren Kopf auf die Schulter der anderen lehnt (*Courage Sonderheft* 1980: 71, 75). Jenseits der Thematik „Psychiatrie“ lässt sich dieses Sonderheft auch als eine Zusammenstellung von Fotografien und Grafiken von Künstlerinnen auffassen, die nicht zwischen psychiatrieerfahrenen und nicht-erfahrenen Künstlerinnen unterschied und auch ohne die Lektüre der Texte im Sinne eines Bildbuchs funktionierte. Somit durchbrach das Heft die verengende Sichtweise, die Werke psychiatrieerfahrener Künstler*innen als Ausdruck ihrer Krankheit zu interpretieren.

## Einbettung in einen psychiatriekritischen und feministischen Kontext

Die *Courage*-Frauen setzten die eingesandten Werke nicht nur über ihre Anordnung und Überschriften in einen feministischen und psychiatriekritischen Kontext, sondern auch durch (eigene) theoretische Reflexionen, Recherchen sowie die Generierung eines feministischen Psy-Wissenskanons. So steuerte beispielsweise die *Courage*-Frau Barbara Duden gemeinsam mit Isabelle Schatten (1978) einen medizinhistorischen Aufsatz zur Hysterie bei – ein in der Frauenbewegung verbreitetes Thema, galt die Hysterie doch als den gesellschaftlichen Verhältnissen geschuldeter Akt der Verweigerung von Frauen gegenüber patriarchalen Zumutungen (ebd.).^46^

Für ihre eigenen Recherchen über Frauen in der Psychiatrie und den Einsatz von Elektroschocks griffen die *Courage*-Frauen zum Teil auf Material der „Kommission für Verstöße der Psychiatrie gegen Menschenrechte e. V.“ in München zurück. Bereits der erste Text, den die *Courage* im März 1978 über die Erlebnisse einer Frau mit der Psychiatrie veröffentlichte (Z[urmühlen] 1978), basierte auf Material, das die *Courage –* offenbar auf eigene Anfrage – von der Kommission erhalten hatte.^47^ Diese war 1972 erstmals öffentlich in Erscheinung getreten und wurde von Scientology unterstützt.^48^ Scientology erklärte die eigene psychiatriekritische Haltung mit Narrativen aus der Tradition antisemitischer Verschwörungstheorien, in die sie ihre Kritik an Elektroschocks und präfrontaler Lobotomie einarbeitete.^49^ In den mir vorliegenden Veröffentlichungen erwähnte die Kommission diese Narrative nicht, zog aber Vergleiche zur Zeit des Nationalsozialismus. Ihr Vorgehen bestand darin, bestimmte psychiatrische Praktiken anzuprangern und Einzelfälle in den Vordergrund zu stellen.^50^ Die Verlautbarungen der Kommission wurden auch von verschiedenen Tageszeitungen aufgegriffen und weiterverbreitet. Dabei wurde die Kommission mit vollständigem Namen und Sitz in München erwähnt, nicht jedoch ihre Beziehung zu Scientology.^51^ Dadurch konnte bei den Leser*innen durchaus der Eindruck entstehen, es handle sich um eine staatliche Einrichtung. In ihrer unkritischen Bezugnahme auf die Kommission stand die *Courage*-Redaktion also keineswegs allein da. Die Nähe der Kommission zu Scientology bedeutete auch nicht, dass die geschilderten Fälle erfunden waren. Allerdings mutet es seltsam an, wenn eine feministische Zeitschriftenredaktion mit ihrer Kritik an patriarchalen Machtstrukturen weiterhin Material der Kommission nutzte, auch nachdem sie über deren Verbindung zu Scientology informiert worden war (P[logstedt]^1980^; *Courage Sonderheft* 1980: 4, 46f.).^52^ Zudem gab die Courage die Adresse der Kommission als Anlaufstelle bei Beschwerden gegen die Psychiatrie an, ohne auf deren Bezug zu Scientology hinzuweisen (*Courage Sonderheft* 1980: 78).^53^

**Abb. 2:**
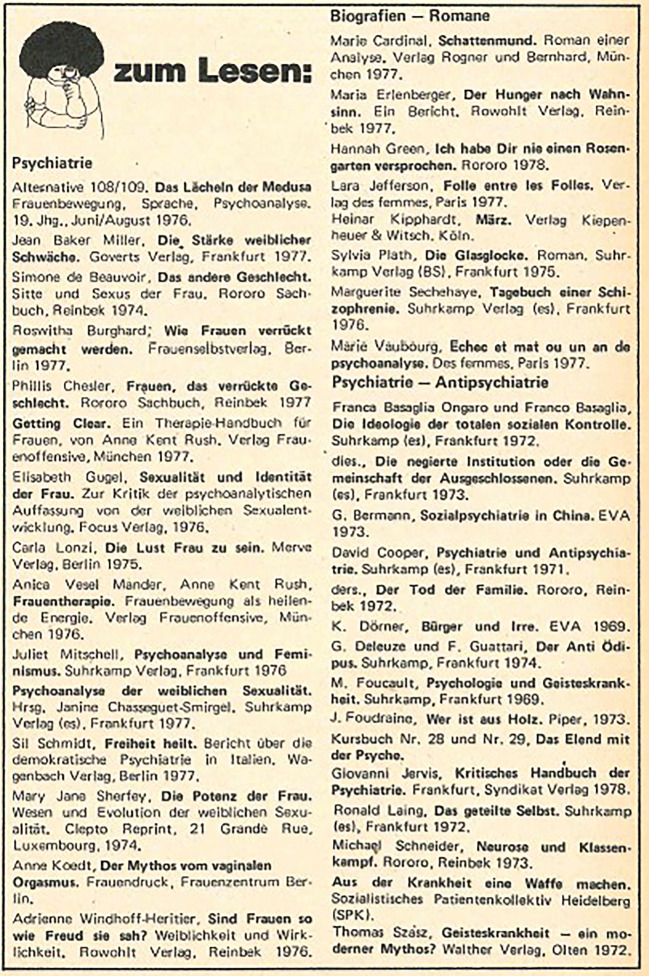
Psy-Kanon in der *Courage* 1978, Heft 4. Bildnachweis: *Courage. berliner frauenzeitung* (1978 3 (4): 47)

Auf den ersten Blick nicht so auffällig, aber für die Generierung eines feministischen Psy-Wissenskanons – so meine These – durchaus bedeutsam waren Literaturlisten in Verbindung mit Buchrezensionen und Verlagsanzeigen. Bereits in ihrem zweiten Schwerpunktheft vom April 1978 veröffentlichte die *Courage*-Redaktion eine zweispaltige, schwarz umrahmte Literaturliste, die ungefähr zwei Drittel der Seite einnahm (*Courage* 1978 (4): 47). Sie war betitelt mit einer Frau in Denkerpose und abstrakter Afro-Frisur sowie den Worten „zum Lesen:“ (Abb. 2). Der Abdruck einer Literaturliste erinnert an akademische Gepflogenheiten und verweist auf die universitäre Herkunft vieler *Courage*-Macherinnen. Die dort gelisteten Bücher können sowohl als Ergänzungen als auch als Werkzeuge zur Analyse der Erfahrungsberichte interpretiert werden sowie als Bedürfnis, die eigenen Handlungen theoretisch zu legitimieren. Die Verwendung eines Bildes einer Frau mit Afro samt Referenz auf die Black-Power-Bewegung kann wiederum als den Büchern zugeschriebenes emanzipatorisches und revolutionäres Potenzial gedeutet werden (Dorestal 2017), Werke Schwarzer Feministinnen waren in der Literaturliste jedoch nicht vertreten.

Die Liste der *Courage*-Frauen deckt eine Bandbreite von Themen und Büchern ab, die bereits in ihrer Zusammenstellung den multiperspektivischen Zugang zum psychiatrischen Feld abbildet. Neben feministischen Werken wie den bereits erwähnten Büchern von Chesler (1974)^54^ und Burgard (1977), Auseinandersetzungen über weibliche Sexualität und Psychoanalyse (*Courage* 1978 [4]: 47)^55^ und den Standardwerken zur feministischen Therapie von Anne Kent Rush und Anica Vesel Mander (Mander & Rush 1976; Rush 1977)^56^ zählten dazu auch die Klassiker der Antipsychiatrie beziehungsweise Psychiatriekritik sowie biografische, literarische und dramaturgische Bearbeitungen von Psychiatrie- und Therapieerfahrungen. In diesen Bearbeitungen kamen nicht nur (fiktionale) Betroffene im Sinne von Expert*innen in eigener Sache zu Wort. Vielmehr ermöglichten sie die Eröffnung einer weiteren Ebene, nämlich psychische Alterität als radikalen Gegenentwurf zu einer entfremdeten bürgerlichen Gesellschaft zu begreifen. Die hier skizzierte Bandbreite an Themen und Zugängen kann zum einen als Versuch gewertet werden, das psychische Leid von Frauen nicht auf isolierte Krankheiten zu reduzieren, sondern sie in den größeren Kontext einer auf sexistischer Diskriminierung beruhenden Gesellschaft zu stellen. Gleichzeitig steht der Kanon auch für die Auffassung, „Wahnsinn“ als gesellschaftsveränderndes Potenzial zu begreifen.

Die Literaturliste sollte in der *Courage* nicht die letzte bleiben. Zwei Jahre später veröffentlichte das Team der Zeitschrift eine erneute Referenzliste empfohlener Lektüre zur Psychiatrie (*Courage Sonderheft* 1980: 72). Bemerkenswerterweise deckte diese aber nicht mehr dieselbe Bandbreite ab, obwohl sie in etwa dieselbe Anzahl an Titeln enthielt. So enthielt die zweite Liste keine sich als sozialistisch oder marxistisch verstehenden Ansätze mehr. Auch die Titel mit Auseinandersetzungen über „weibliche Sexualität“ waren stark reduziert; ebenso die psychiatriekritischen Klassiker. Demgegenüber hatte die Zahl der Autobiografien und Therapieberichte zugenommen. An der zweiten Liste lässt sich vor allem die Bedeutungszunahme feministischer Therapie ablesen.

Neben den beiden Literaturlisten trug auch der Einsatz von Werbung zur Entstehung eines entsprechenden Wissenskanons bei. Die *Courage* druckte nur Werbung ab, die in Einklang mit ihren politischen Positionen war. Dies bedeutete vor allem Werbung für andere Frauenprojekte, aber auch für Bücher und Zeitschriften, die aus nicht dezidiert feministischen Verlagen stammten, deren Themen aber für interessant befunden wurden. So warb beispielsweise das populärwissenschaftliche Magazin *Psychologie heute* im erwähnten Sonderheft von 1980 mit einer ganzseitigen Anzeige. Darin präsentierte sich die Zeitschrift als „Forum für die Diskussion innerhalb der Frauenbewegung“ und verwies unter anderem auf die Titelgeschichte in ihrer Ausgabe vom Juni 1980 zum Thema „Frauenliebe“ (*Courage Sonderheft* 1980: 31). Auch der Rowohlt-Verlag warb in der *Courage* für zwei Bücher von Maria Erlenberger. Im Falle dieser Werbung konnten die Leser*innen eine direkte Beziehung herstellen zwischen Erlenbergers (1977) in der Anzeige aufgeführtem Bericht *Der Hunger nach Wahnsinn* mit dem nebenstehenden Gedicht „Die ersten Tage in der Geschlossenen“ von Anja Rieger (*Courage*-*Sonderheft* 1980: 25). Bereits in vorherigen Ausgaben der *Courage *hatte es entsprechende Anzeigen gegeben, zudem hatte die Zeitschrift einige der in den Literaturlisten aufgeführten Bücher ausführlich rezensiert (Lorez 1977; Schöfthaler & Zurmühl 1977). Gerade durch diese Wiederholungen und Referenzen entstand ein psy-feministischer Literaturkanon.

Zusammenfassend lässt sich sagen, dass die *Courage* im Lauf weniger Jahre einen Literaturkanon entwickelte, der medizinisch geprägten psychopathologischen Krankheitskonzepten nur wenig Raum bot. Stattdessen wurden psychische Leiden und psychische Alteritäten in eine soziale Relation zur Gesellschaft gesetzt – mit einem speziellen Fokus auf der Lage der Frau. Hierzu lieferten Psychologie und Psychoanalyse trotz der Kritik an deren Grundlagen wichtige Instrumente. Die Fokussierung auf das Soziale zog zugleich eine Dekonstruktion von Geschlecht als naturalisierter Differenzkategorie nach sich. Die Konzentration auf Frauen als soziale Gruppe barg allerdings auch die Gefahr, Geschlecht unter anderen Vorzeichen erneut festzuschreiben.

## Auswirkungen und Diskussion

Der partizipative Ansatz bei der Herstellung der Zeitschriften ermöglichte die Generierung eines psy-feministischen Wissens, das auch die Erfahrungen psychiatrieerfahrener Frauen miteinbezog. Damit nahm die *Courage* im Ansatz eine Entwicklung vorweg, die antipsychiatrische Gruppen wie die „Irrenoffensive“ in den 1980er Jahren einforderten (Stöckle 1983), dabei aber den Genderaspekt nur punktuell thematisierten. Die *Courage *verfolgte zwar einen psychiatriekritischen, jedoch keinen antipsychiatrischen oder dezidiert sozialpsychiatrischen Kurs. So wurden psychiatrische Diagnosen in der Zeitschrift zum Teil abgelehnt, zum Teil aber auch übernommen oder angeeignet. Durch diese flexible Rezeption umgingen die *Courage*-Frauen die damals verbreitete Polarisierung zwischen diesen beiden Ansätzen.^57^ Gerade ihre multiperspektivische und multimodale Herangehensweise, die sowohl künstlerische Bearbeitungen als auch gesellschaftskritische Auseinandersetzungen miteinschloss, stellte die Psychiatrie als medizinische Disziplin mit der alleinigen Definitionsgewalt über „Normalität“ und „Wahnsinn“ infrage. Darüber hinaus setzte die *Courage *mittels Überschriften, Text-Bild-Anordnungen und theoretischen Zugängen die Erfahrungsberichte in einen dezidiert feministischen und psychiatriekritischen Kontext. Zudem bildete sich in der *Courage* die enge Verknüpfung von Frauenbewegung, Selbsthilfe und Psychologie ab – bot sie doch feministische Selbsterfahrungsgruppen und Therapie als Lösungswege an und nicht Aktionen gegen die Institution Psychiatrie, wie sie beispielsweise die „Sozialistische Selbsthilfe Köln“ (SSK) seit Mitte der 1970er Jahre durchführte und dokumentierte.^58^

Gleichzeitig implementierten die *Courage*-Macherinnen einen Stil, der das Erleben von psychischem Leid und psychischer Alterität in den Vordergrund stellte. Der erfahrungsbasierte und partizipative Ansatz hatte jedoch auch seine Grenzen. Diese lagen wesentlich im Medium und dessen Herstellung. Um zum öffentlichen Diskurs beitragen zu können, mussten potenzielle Autorinnen die *Courage* kennen. Darüber hinaus mussten sie in der Lage sein, ihre Erfahrungen zu verschriftlichen und einzusenden. Um gedruckt zu werden, mussten diese Niederschriften wiederum von den *Courage*-Frauen für akkurat, interessant und lesbar befunden werden. Frauen, die diese Bedingungen nicht erfüllten, blieben von der Generierung psy-feministischen Wissens in der Zeitschrift ausgeschlossen.

## Footnotes

1 Ich verwende hier den Terminus Frauen ohne Gender-Asterisk, weil „Frauen“ (ab den 1980er Jahren dann zunehmend „Frauen/Lesben“) in meinem Untersuchungszeitraum die gängige Selbstbezeichnung in der autonomen Frauenbewegung in der Bundesrepublik war.

2 Die Termini „Psychiatrieerfahrung“ bzw. „psychiatrieerfahren“ wurden in der *Courage *nicht verwendet. Ich benutze diese Begriffe, um den – meist als problematisch empfundenen – Aufenthalt in der Psychiatrie als verbindendes Element dieser Frauen deutlich zu machen und nicht eine psy-medizinische Diagnose.

3 Vgl. u. a. zu Plath: Zöfel (1979); zu Zürn: Henry (1984); zu Perkins Gilman: Perkins Gilman (1978).

4 Vgl. u. a. Ane et al. (1977); Bindseil (1978); Anonym (1982a); Seifert (1985).

5 Auch andere feministische Zeitschriften griffen das Thema Frauen in der Psychiatrie auf, aber nicht in dieser Breite. Außerdem verwendeten sie als Format Interviews, literaturwissenschaftliche Abhandlungen oder Reportagen. Vgl. *Emma* 1979 (2); *Wissenschaft und Zärtlichkeit* 1981 (10/11); *Lesbenstich* 1982 (1).

6 Ich verwende Psy als Überbegriff zur Beschreibung eines psycho-sozialen und psycho-mentalen Feldes, das sowohl die Disziplinen Psychiatrie, Psychologie und Psychotherapie umfasst als auch die Negation und Kritik an den ihnen zugrunde liegenden Konzepten, Institutionen und Praktiken. Darüber hinaus beinhaltet Psy auch edukative und alltägliche Praktiken sowie alternative Behandlungsformen des Psychischen.

7 Da die *Courage* zwischen Redakteurinnen und Layout-Frauen unterschied, benutze ich die Begriffe *Courage*-Frauen oder *Courage*-Macherinnen, wenn ich von allen am direkten Herstellungsprozess der Zeitschrift beteiligten Frauen spreche.

8 Zur bereits zeitgenössischen Problematisierung der Priorisierung von Erfahrung in Selbsterfahrungsgruppen, die – so der Vorwurf der *Schwarzen Botin* – nur zu einem Feststecken im eigenen Leiden führe vgl. Lux (2022: 215–238). Scott (1991) kritisierte, dass Erfahrungen zwar repressive Mechanismen sichtbar machen können, dabei aber die Analyse der inneren Logik und des Funktionierens von Machttechniken auf der Strecke bleibe. Eine Darstellung der zu Beginn der 1990er Jahre geführten Debatte über Erfahrung in den Frauen- und Geschlechterstudien findet sich in Bos et al. (2004). Vgl. auch Schlichters (2000) Kritik an der Autorisierung weiblicher Stimmen als Basis feministischer Psychiatriekritik.

9 Alexander von Schwerin (2022: 537) definiert Gegenwissen als „gesellschaftliche Absetzbewegung“, die sowohl die Kritik an einer als hegemonial verstandenen Wissenschaft, Medizin und Technik umfasst als auch die Generierung eigener Wissensinhalte und -formen, den Aufbau eigener Strukturen der Wissensgenerierung sowie das Einüben spezieller epistemischer Praxen. Zum Begriff des Gegenwissens und zur Funktion, die Medien bei seiner Erzeugung einnehmen, vgl. auch Stadler et al. 2020.

10 Zur Zeitschriftenproduktion der autonomen Frauenbewegung in deutschsprachigen Ländern (vgl. u. a. Weinel 1984; Schneegass 1993; Deininger 2004; Notz 2007; Susemichel et al. 2008; Vukadinović 2021; Lux 2022; Schallner 2022).

11 In der Aufhebung der Distanz zwischen Zeitschriftenmacher*innen und Leser*innen sieht Reichardt (2014: 238, 280–284) ein wichtiges Merkmal der Alternativpresse. Die Macherinnen der *Schwarzen Botin*, die sich immer wieder gegen die Priorisierung vermeintlich weiblicher Erfahrungen wendeten, verzichteten wiederum auf den Abdruck von Interna.

12 Für diesen Aufsatz wurden folgende Zeitschriften aus den 1970er und 1980er Jahren vollständig gesichtet: *Courage, Emma, Die Schwarze Botin, Hamburger Frauenzeitung, Lesbenstich, Clio, Streit, Anagan, Hexengewitter *sowie einzelne Ausgaben von *Wissenschaft und Zärtlichkeit, Frau und Film, Mamas Pfirsiche, Protokolle – Informationsdienst für Frauen, Beiträge zur Feministischen Theorie und Praxis, Schreiben, Die kleine Hexe, Lila Distel, Frankfurter Frauenzeitung, Frauenoffensive Journal, nebenwiderspruch, Frauenzeitung – Frauen gemeinsam sind stark, schamlos, Xanthippe*. Hilfreich waren außerdem das APO-Archiv der Freien Universität Berlin, das Flugblätter und Zeitschriften zur Frauenbewegung enthält, sowie die umfangreichen Zeitschriften- und (graue) Literatursammlungen im Frauen-Media-Turm in Köln.

13 So fand 1973 im West-Berliner Kino Arsenal das erste internationale Frauenfilmseminar statt. 1974 gründete die Filmemacherin Helke Sander die Zeitschrift *Frau und Film* und 1977 eröffnete das erste Frauenkino in West-Berlin (Altbach 1984). In Hamburg existierte mit „Bildwechsel“ ein Frauenmedienladen, der es sich zur Aufgabe gemacht hatte, eigene Video- und Fotoprojekte zu machen, Filme und Videos vorzuführen, letztere zu verleihen und andere Frauen in ihrer Arbeit mit Druck, Video und Fotografie zu unterstützen. Vgl. die Broschüre „hamburger frauengruppen stellen sich vor“, S. 6f., in FFBIZ, A Rep. 400 BRD 20 (3), o. p.

14 Zur alternativen Ökonomie in Frauenbetrieben vgl. Plogstedt (2006). Zum Verhältnis von Frauenbewegung und alternativem Milieu vgl. Lenz (2010).

15 Die erste periodisch erscheinende Zeitschrift war die vom sozialistischen Frauenbund Westberlin 1970 zum ersten Mal publizierte *Pelagea*. Vgl. Weinel (1984: 40).

16 Aufgrund ihrer Bedeutung für die bundesrepublikanische Frauenbewegung zählt Weinel auch die Zeitschrift *Der Feminist *dazu, in der Männer ebenfalls Artikel schrieben und die sich explizit auch an Männer richtete, sowie die hauptsächlich in Bern herausgegebene Zeitschrift *Die Hexenpresse*, die aber auch eine Redaktionsadresse in der Bundesrepublik hatte. Vgl. Weinel (1984: 46).

17 Reichardt (2014: 245) spricht von 383 feministische Zeitschriften für die Zeit zwischen 1970 und 1980 und von 40 Zeitschriften zu Beginn der 1980er Jahre. Schallner (2022) verweist hingegen auf 100 Neugründungen zwischen 1980 und 1985.

18 Rasch (1983), in: FFBIZ, A Rep. 400 BRD 20 (3), o. p.

19 Für eine genaue Auflistung vgl. Weinel (1984: 51, 63–65, 105–122). Weinel differenziert zwischen acht überregionalen und 52 regionalen Zeitschriften, 35 Zielgruppenzeitschriften, 16 themenorientierten und elf theoretischen Zeitschriften sowie elf Informationsdiensten. Zu den verschiedenen Zeitschriften vgl. auch Schallner (2022).

20 Reader der Zeitungstreffen finden sich im Archiv des Hamburger Instituts für Sozialforschung (HIS), SBe_181 HFZ, „historisch“ 1981.

21 Dies war zum einen das Motto eines Autorinnentreffens im November 1976 in West-Berlin (Kaever 1977) und zum anderen der Name einer lokalen West-Berliner Arbeitsgruppe schreibender Frauen (Petersen & Garbe 1978).

22 Das erste Treffen schreibender Frauen organisierte der Verlag Frauenoffensive 1976 in München; vgl. die Beiträge in *Frauenoffenisve Journal Nr.* 5 (1976) sowie die Kritik an diesen Beiträgen in der *Schwarzen Botin *(Anonym 1976). Zum Autorinnentreffen im gleichen Jahr in West-Berlin vgl. u. a. Kaever (1977). Zum Treffen der Schreibenden Frauen in Köln 1977, das sich als Fortsetzung des Münchener Treffens verstand, vgl. Moszkovicz (1977); Anonym (1978b). Zum 5. Treffen Schreibender Frauen 1984 in Bremen vgl. Venske 1988.

23 Auch Männer lasen die Courage (Plogstedt & Arnholdt 1978). Abgesehen von Leserbriefen konnten sie aber keine Beiträge einreichen.

24 Zu einer kritischen Diskussion des Begriffes Gegenöffentlichkeit vgl. Wimmer (2007).

25 Vgl. z. B. die regelmäßige Werbung für oktober druck oder viva frauendruck in der *Courage*.

26 Fixogum ist ein Klebstoff, der den Vorteil hat, für eine gewisse Zeit verschiebbar zu bleiben und sich rückstandsfrei entfernen zu lassen. Letraset waren Anreibebuchstaben, die mit einem harten Stift oder Anreibelöffel aufgebracht wurden.

27 Vgl. hierzu auch das Foto bei Mehl (2021).

28 Was das bedeutete, wurde im Bericht über das Frauenzeitungstreffen nicht weiter ausgeführt.

29 Tatbestand schreiblich. Frauenzeitungstreffen, Berlin 1988, in HIS, SBe_181_HFZ, „historisch“ 1981, o. p.

30 Plogstedt verlor aufgrund ihrer Mitgliedschaft in der Gruppe Internationaler Marxisten (GIM) ihren Job an der Freien Universität Berlin (Schneegass 1993: 78f.).

31 Im Novemberheft der *Courage* von 1979 mit dem Schwerpunkt „Der verdammte Alkohol“ (vgl. auch den Beitrag von Viola Balz in diesem Heft) spielt die Psychiatrie hingegen so gut wie keine Rolle.

32 Es handelte sich um Marion Ballé, die Germanistinnen Christel Dormagen, Conny Döhring und Sabine Zurmühl, die auch freie Autorin beim Westdeutschen Rundfunk (WDR) sowie beim Rundfunk im amerikanischen Sektor (RIAS) war, Birgit Klarner, die Fotografin Birgit Kleber, die Psychologin Christa Müller, die Sozialwissenschaftlerin Sibylle Plogstedt, Barbara Pörner, die Jura studiert hatte, die im Druckhandwerk ausgebildete Ingrid Schulte, Gesine Strempel, Olga-Maria Werner sowie Barbara Weber. Zu den beruflichen Angaben vgl. Schneegass (1993).

33 Die ehemalige *Courage*-Frau Barbara Duden sprach rückblickend vom Gebot „‚authentisch‘ zu sprechen und zu schreiben“ (Notz 2007: 39). Reichardt (2014) arbeitete das Streben nach Authentizität als zentrales Moment des linksalternativen Milieus heraus, zu dem er auch die Frauenbewegung zählt.

34 Prinzipiell hätten auch Männer unter weiblichem Pseudonym Texte verfassen können.

35 Zur radikalen Psychiatriekritik in der BRD vgl. Beyer (2022).

36 Mitarbeiter*innen psychiatrischer Anstalten kamen außerdem in den Interviewauszügen von Inge Buck (1980) zu Wort.

37 Darüber hinaus fehlte die Gruppe der Angehörigen. Auch sogenannte Gastarbeiterinnen kamen weder als in der Psychiatrie Beschäftigte noch als Insassinnen zu Wort.

38 Zur Vorbildfunktion von Anstaltskunst für surrealistische Künstler*innen vgl. Röske (2009).

39 Mit Ausnahme der Werke Victor Brauers handelte es sich im Sonderheft durchweg um die Arbeiten von Frauen.

40 Für eine zeitgenössische kritische Diskussion des „Hausfrauensyndroms“ vgl. Pross (1979, S. 222–229).

41 Zu feministischen Bildpolitiken vgl. Susemichel (2008).

42 Der Fotoband enthält weder Seitenzahl noch Angaben dazu, wer die einzelnen Fotos geschossen hat oder aus welcher Anstalt sie stammen. Foot (2015) hat anhand der in den Bildern zu sehenden Architektur und späterer Texte die Orte identifiziert. Zum Einfluss Basaglias auf die deutsche Psychiatriereform vgl. Marazia et al. (2020).

43 Offensichtlich war sich auch Basaglia selbst dieser Problematik bewusst (Foot 2015: 21).

44 Ich bedanke mich bei Christof Beyer für den Hinweis auf dieses Buch.

45 *Courage Sonderheft* (1980: 4f., 9, 28ff., 39, 43).

46 Für einen Überblick über die feministische Analyse der Hysterie vgl. Nolte (2003, S. 13–16).

47 Schreiben der Kommission an die *Courage *vom 26.11.1970, in FFBIZ, A Rep. 400 BRD 16.22.13, o. p. Der in der *Courage* beschriebene Fall der Dorothea K. ist auch in der monatlichen Mitteilung der Kommission *Menschenrechte aktuell* vom Dezember 1977 abgedruckt. Vgl. Bundesarchiv (BArch), B 189/35266, o. p.

48 Vgl. ABI-Aktion Bildungsinformation e. V., Stuttgart (Hg.) o. J.: *Die Scientology-Sekte und ihre Tarnorganisationen*, in BArch B 189/35267, o. p.

49 Schreiben des Guardian Office der Scientology Kirchen und Missionen in Deutschland an das Bundesministerium für Jugend, Familie und Gesundheit vom 24.06.1980, in BArch, B 189/22161, o. p. Der Verfasser des Schreibens bezog sich nicht explizit auf jüdische Personen, verwendete aber mehrere antisemitische Codes. Zur Verknüpfung von Antisemitismus und Verschwörungstheorien vgl. Butter (2018, 168f).

50 Vgl. z. B. die von der Kommission für Verstöße der Psychiatrie gegen Menschenrechte herausgegebene Broschüre *Gefahren eines neuen psychiatrischen Holocaust. Phantasien oder schon Realität?*, die auf der gleichnamigen Tagung im November 1979 in München basierte, in BArch B 189/35266.

51 Ein vom Sprecher der Kommission Georg Stoffel verfasster Artikel erschien z. B. in den *Westfälischen Nachrichten* vom 06.08.1977. Abdrucke dieses sowie weiterer Zeitungsartikel, in denen die Kommission genannt wird, finden sich in verschiedenen Publikationen der Kommission in BArch B 189/35266.

52 Schreiben an die *Courage *vom 14.03.1978, in FFBIZ, A Rep. 400 BRD 16.22, o. p.

53 Schreiben der Kommission für Verstöße der Psychiatrie gegen Menschenrechte an die *Courage *vom 20.03.1980, in FFBIZ, A Rep. 400 BRD 16.22.13, o. p.

54 Zur Kritik an Chesler vgl. Schlichter (2000).

55 Zum Verhältnis von Feminismus und Psychoanalyse vgl. Lindhoff (2003).

56 Zur deutschen Adaptation von Rushs und Manders Buch vgl. Ruck et al. (2022).

57 Zur Polarisierung zwischen Sozialpsychiatrie und Antipsychiatrie vgl. Stöckle (1983); Brink (2010).

58 Zur Sozialistischen Selbsthilfe Köln vgl. u. a. Hemmerling (2019).

## References

[CR40] [Goettle, Gabriele] 1976. Schleim oder Nichtschleim, das ist hier die Frage. *Die Schwarze Botin* (1): 4–5.

[CR1] Altbach, Edith Hoshino 1984. The New German Women’s Movement. *Signs* 9 (3): 454–469.

[CR11] Anagan 1984. Anstatt einer Selbstdarstellung. *Anagan* (1): 3.

[CR2] Ane, Christina, Ingeborg, Heidi, Sophinette 1977. Feminismus und Psychoanalyse. *protokolle. informationsdienst für frauen* (16): 4–12.

[CR3] Anonym 1976. Dann spürten wir den bösen Blick der Abstraktion. Rezension des Heftes 5 der Frauenoffensive „Aufständische Kultur“. *Schwarze Botin* 1 (1): 28–30.

[CR4] Anonym 1977. In eigener Sache. *Protokolle. informationsdienst für frauen* (15): 3.

[CR5] Anonym 1978a. In eigener Sache. *Courage. berliner frauenzeitung* 3 (6): 2.

[CR6] Anonym 1978b. Treffen Schreibender Frauen Köln Nov. 77. *Schreiben* 2 (1): 1–3.

[CR7] Anonym 1980a. Hier nimmt der Ekel alles Mitgefühl. *Courage. Sonderheft* 2 (2): 33–35.

[CR8] Anonym 1980b. Wahnideen. *Courage.**Sonderheft* 2 (2): 36–37.

[CR9] Anonym 1982a. Die totgesagte Vagina. *Hexengewitter* (3): 26–32.

[CR10] Anonym 1982b. In eigener Sache. *Courage. aktuelle frauenzeitung* 7 (9): 2.

[CR12] Basaglia, Franco, und Franca Basaglia Ongaro (Hg.) 1969. *Morire di classe. La condizione manicomiale fotografata da Carla Cerati e Gianni Berengo Gardin*. Torino: Giulio Einaudi.

[CR13] Beyer, Christof 2022. Radikale Psychiatriekritik und die Transformation des Anstaltswesens in der Bundesrepublik. In: Wilfried Rudloff, Franz-Werner Kersting, Marc Miquel und Malte Thießen (Hg.). *Ende der Anstalten? Großeinrichtungen, Debatten und Deinstitutionalisierung seit den 1970er Jahren*. Paderborn: Brill Schöningh: 155–173.

[CR14] Bindseil, Ilse 1978. Sie ist hysterisch – er hat die Zwangsneurose. *Courage. aktuelle frauenzeitung* 3 (2): 29–31.

[CR15] Blessing, Annemie 1980. „Ich bin auch mal die Stärkere“. Co-Counselling-Gruppe. *Courage. Sonderheft* 2 (2): 77.

[CR16] Bos, Marguérite, Bettina Vincenz und Tanja Wirz (Hg.) 2004. *Erfahrung: Alles nur Diskurs? Zur Verwendung des Erfahrungsbegriffes in der Geschlechtergeschichte*. Zürich: Chronos.

[CR17] Brink, Cornelia 2010. *Grenzen der Anstalt. Psychiatrie und Gesellschaft in Deutschland 1860–1980*. Göttingen: Wallstein.

[CR18] Buck, Inge 1980. Wir haben fast alles Hausfrauen. Interviews. Auszug aus einer Sendung von Radio Bremen. *Courage. Sonderheft* 2 (2): 4–11.

[CR19] Burgard, Roswitha 1977. *Wie Frauen „verrückt“ gemacht werden*. Berlin-West: Frauenselbstverlag.

[CR20] Butter, Michael 2018. „Nichts ist, wie es scheint“. Über Verschwörungstheorien. Frankfurt am Main: Suhrkamp.

[CR21] Chesler, Phyllis 1974. *Frauen – das verrückte Geschlecht? Mit einem Vorwort von Alice Schwarzer.* Reinbek: Rowohlt.

[CR22] Cibach, Anke 1980. „Danke, ja gut“. *Courage. Sonderheft* 2 (2): 69–70.

[CR23] Cornelia 1980. Die Psychiatrie ist der größte Puff. *Courage. Sonderheft* 2 (2): 32.

[CR24] *Courage. berliner frauenzeitung *1978 3 (4).

[CR25] Courage-Redaktion 1976. In eigener Sache. *Courage. berliner frauenzeitung* 1 (0): 2.

[CR26] *Courage Sonderheft* 1980 2 (2).

[CR27] Davis, Kathy 2007. *The Making of Our Bodies, Ourselves. How Feminism Travels across Borders*. Durham: Duke University Press.

[CR28] Deininger, Kristina 2004. Feministische Presse in der Bundesrepublik Deutschland: Emma und die feministische Öffentlichkeit. Magistraarbeit, Freie Universität Berlin.

[CR29] Die TUBFF-Frauen 1980. „Hier bin ich die Frau der Stunde“. *Courage. Sonderheft* 2 (2): 71–73.

[CR30] Dietze, Gabriele 1979. Vorwort. „Überwindung der Sprachlosigkeit“. In: dies. (Hg.). *Die Überwindung der Sprachlosigkeit. Texte aus der neuen Frauenbewegung*. Darmstadt: Luchterhand: 7–21.

[CR31] Discher, Marion 1980. „Bist wohl von der Elitestation“. *Courage. Sonderheft* 2 (2): 20–21.

[CR32] Dorestal, Philipp 2017. Dressing the Black Body. Mode, Hairstyle und Schwarzsein in den USA – von den 1970er Jahren bis zu Barack Obama. *Zeithistorische Forschungen/Studies in Contemporary History*, Online-Ausgabe. 10.14765/zzf.dok.4.971.

[CR33] Dormagen, Christel, Sabine Zurmühl, Ingrid Schulte und Sibylle Plogstedt 1982. In eigener Sache. *Courage. aktuelle frauenzeitung* 2: 58–59.

[CR34] Duden, Barbara und Isabelle Schatten 1978. Die Gebärmutter – Das hungrige Tier. Zur Geschichte der Hysterie. *Courage. berliner frauenzeitung* 3 (3): 19–23.

[CR35] E. 1980. Liebe Katarina. *Courage. Sonderheft* 2 (2): 61.

[CR36] Eine 18-jährige Schülerin 1980. Eine feste Wand zum Dagegenrennen. *Courage. Sonderhe*ft 2 (2): 26–27.

[CR103] *Emma* 1979 (2).

[CR37] Erlenberger, Maria 1977. *Der Hunger nach Wahnsinn. Ein Bericht*. Reinbek: Rowohlt.

[CR38] Foot, John 2015. Photography and radical psychiatry in Italy in the 1960s. The case of the photobook Morire di classe (1969). *History of Psychiatry* 26 (1): 19–35. 25698683 10.1177/0957154X14550136 PMC4361699

[CR39] Friedan, Betty 1966. *Der Weiblichkeitswahn oder Die Mystifizierung der Frau*. Reinbek: Rowohlt.

[CR41] Goffman, Erving 1973. *Asyle. Über die soziale Situation psychiatrischer Patienten und anderer Insassen*. Frankfurt am Main: Suhrkamp.

[CR42] Heiliger, Henriette 1980. Leserinnenbrief: Sonderheft 2. *Courage. aktuelle frauenzeitung* 5 (7): 58.

[CR43] Hemmerling, Jutta 2019. *Protestaktionen gegen Missstände in der Psychiatrie am Beispiel des Vereins Sozialistische Selbsthilfe Köln in den 1970er und 1980er Jahren*. Göttingen: Cuvillier.

[CR44] Henry, Ruth 1984. Unica Zürn. *Emma. Zeitschrift für Frauen von Frauen* (8): 38–40.

[CR45] K. K. 1980. Du hast keine Chance. *Courage. Sonderheft* 2 (2): 19.

[CR46] Kaever, Roswitha 1977. Schreib das auf, Frau! Formen, Formeln, Formulierungen eines Autorinnentreffens, das an einem sonnigen Wochenende im November stattfand. *Schwarze Botin* 2 (2): 28–29.

[CR47] Katharina 1980. „Angst sind meine Wurzeln“. *Courage. Sonderheft* 2 (2): 60.

[CR48] Klarner, Birgit 1980. In eigener Sache. *Courage. aktuelle frauenzeitung *5 (7): 2.

[CR49] Klarner, Birgit 1982. Übers Feministeln. Ein Auszug. *Courage. aktuelle frauenzeitung *7 (1): 55–56.

[CR50] Kwaschik, Anne 2023. „We Witches“. Knowledge Wars, Experience and Spirituality in the Women’s Movement During the 1970s. *NTM Zeitschrift für Geschichte der Wissenschaften, Technik und Medizin *31 (2): 171–199. 10.1007/s00048-023-00359-w PMC10271903 37222765

[CR51] Lenz, Ilse 2010. Das Private ist politisch!? Zum Verhältnis von Frauenbewegung und alternativem Milieu. In: Detlef Siegfried und Sven Reichardt (Hg.). *Das Alternative Milieu. Antibürgerlicher Lebensstil und linke Politik in der Bundesrepublik Deutschland und Europa 1968–1983*. Göttingen: Wallstein: 375–404.

[CR101] *Lesbenstich* 1982 (1).

[CR52] Lindhoff, Lena 2003. *Einführung in die feministische Literaturtheorie*. 2. Aufl. Stuttgart: J. B. Metzler.

[CR53] Lorez, Gudula 1977. Getting Clear. Ein Therapie-Handbuch für Frauen. *Courage. berliner frauenzeitung* 2 (10): 36–37.

[CR54] Lux, Katharina 2022. *Kritik und Konflikt. Die Zeitschrift Die Schwarze Botin in der autonomen Frauenbewegung.* Wien: mandelbaum kritik & utopie.

[CR55] M., Carola 1978. Als Krankenschwester in der Psychiatrie: beobachten, beaufsichtigen, kontrollieren. *Courage. aktuelle frauenzeitung* 3 (4): 43–44.

[CR56] Mander, Anica Vesel und Anne Kent Rush 1976. *Frauentherapie*. München: Frauenoffensive.

[CR57] Marazia, Chantal, Heiner Fangerau, Thomas Becker und Felicitas Söhner 2020. „Visions of another world“. Franco Basaglia and German reform. In: Tom Burns und John Foot (Hg.). *Basaglia’s International Legacy. From Asylum to Community*. Oxford: Oxford University Press: 227–244.

[CR58] Mark, Mary Ellen und Karen Folger Jacobs 1979. *Ward 21*. New York: Simon and Schuster.

[CR59] Mehl, Friederike 2021. Die Zeitschrift *Courage*. Digitales Deutsches Frauenarchiv. URL: https://www.digitales-deutsches-frauenarchiv.de/themen/die-zeitschrift-courage#?id=17720ffbiz_1&open=&c=&m=&s=&cv=&xywh=-79%2C-388%2C2247%2C2238 (08.10.2024).

[CR60] Miller, Nadine 1980. An meinen Therapeuten. *Courage. Sonderheft* 2 (2): 67–68.

[CR61] Moszkovicz, Ela 1977. Schreibende Frauen. *Courage. berliner frauenzeitung* 2 (12): 49.

[CR62] Nolte, Karen 2003. *Gelebte Hysterie: Erfahrung, Eigensinn und psychiatrische Diskurse im Anstaltsalltag um 1900*. Frankfurt am Main: Campus.

[CR63] Notz, Gisela 2007. Courage – Wie es begann, was daraus wurde und was geblieben ist. In: dies. (Hg.). *Als die Frauenbewegung noch Courage hatte. Die „Berliner Frauenzeitung Courage“ und die autonomen Frauenbewegungen der 1970er und 1980er Jahre*. Bonn: Friedrich-Ebert-Stiftung: 23–56.

[CR64] Perkins Gilman, Charlotte 1978. Die gelbe Tapete. *Courage. aktuelle Frauenzeitung* 3 (3): 11–18.

[CR65] Petersen, Karin und Christine Garbe (mithilfe von Gesprächen mit Stefani Majer und vielen anderen) 1978. Die gelben Socken und ihre Grenzen. *Courage. berliner frauenzeitung* 3 (7): 28–29.

[CR66] Petersen, Margret. 1978. „Ja, Liebes, so eine Mutti hast du“. *Courage. aktuelle frauenzeitung* 3 (3): 7–10.

[CR67] P[logstedt], S[ibylle] 1980. Eine ganz zauberhafte Wirkung. Ärzte über E‑Schocks. *Courage. Sonderheft* 2 (2): 45–46.

[CR68] Plogstedt, Sibylle 2006. *Frauenbetriebe. Vom Kollektiv zur Einzelunternehmerin.* Königstein (Taunus): Ulrike Helmer.

[CR69] Plogstedt, Sibylle und Monika Arnholdt 1978. Wer sind die Courage-Leserinnen? *Courage. aktuelle frauenzeitung* 3 (11): 22–29.

[CR70] Pross, Helge 1979. *Die Wirklichkeit der Hausfrau*. 3. Aufl. Reinbek: Rowohlt.

[CR71] PSIFF 1980. „… daß nicht alles so aussichtlos ist.“ PSIFF-Gespräche. *Courage. Sonderheft* 2 (2): 74–76.

[CR72] Rasch, Ute 1983. Neu in Düsseldorf: „Was machst du abends?“ Ein Ratgeber nur für Frauen. *Neue Rhein Zeitung* 26.3.1983.

[CR73] Rebecca 1980. Die Totenstille auf der Station. *Courage. Sonderheft* 2 (2): 14–18.

[CR74] Reichardt, Sven 2014. *Authentizität und Gemeinschaft. Linksalternatives Leben in den siebziger und frühen achtziger Jahren.* Berlin: Suhrkamp.

[CR75] Reumschüssel-Wienert, Christian 2021. *Psychiatriereform in der Bundesrepublik Deutschland. Eine Chronik der Sozialpsychiatrie und ihres Verbandes – der DGSP*. Bielefeld: Transcript.

[CR76] Röske, Thomas 2009. Inspiration und unerreichtes Vorbild – L’art des fous und Surrealismus. In: ders. und Ingrid von Beyme (Hg.). *Surrealismus und Wahnsinn. Surrealism and Madness*. Heidelberg: Das Wunderhorn: 9–19.

[CR77] Ruck, Nora, Vera Luckgei, Barbara Rothmüller, Nina Franke und Emelie Rack 2022. Psychologization in and through the women’s movement: A transnational history of the psychologization of consciousness-raising in the German-speaking countries and the United States. *Journal of The History of the Behavioaral Sciences* 58 (3): 269–290. 10.1002/jhbs.22187 PMC9542099 35239977

[CR78] Rush, Anne Kent 1977. *Getting Clear. Ein Therapie-Handbuch für Frauen*. München: Frauenoffensive.

[CR79] S., Doro 1978. Bericht einer Ärztin: Wer geht schon in der Psychiatrie arbeiten? *Courage. aktuelle frauenzeitung* 3 (4): 44–47.

[CR80] Schalk, Gisela 1988. Schreiben befreit oder Von der Methode zum Inhalt. *Schreiben* 11 (32): 78–84.

[CR81] Schallner, Berit 2022. Neue Zöpfe und Das Lächeln der Medusa: Zeitschriften der Neuen Frauenbewegung in der BRD der 1970er und 1980er Jahre. *Digitales Deutsches Frauenarchiv*. URL: https://www.digitales-deutsches-frauenarchiv.de/themen/zeitschriften-der-neuen-frauenbewegung-der-brd-der-1970er-und-1980er (08.10.2024).

[CR82] Schlichter, Annette 2000. *Die Figur der verrückten Frau. Weiblicher Wahnsinn als Kategorie der feministischen Repräsentationskritik*. Tübingen: edition eskord.

[CR83] Schneegass, Beate 1993. Feminismus im Brennpunkt. Die Frauenzeitung Courage und ihre Mütter. Geschichte – Entwicklung – Wirkung. In: Angelika Oettinger und dies. (Hg.). *Gebraucht, gebremst … gefördert. Frauen und Politik in Charlottenburg nach 1945*. Berlin: Edition Hentrich: 74–112.

[CR84] Schöfthaler, Else und Sabine Zurmühl 1977. Roman einer Analyse. Schattenmund. *Courage. berliner frauenzeitung* 2 (5): 21.

[CR85] Schwerin, Alexander von 2022. Gegenwissen. Die Neuen Sozialen Bewegungen in der Bundesrepublik und die Grundlagen ihrer Wirkung. *NTM Zeitschrift für Geschichte der Wissenschaften, Technik und Medizin *30 (4): 529–540. 10.1007/s00048-022-00349-4 PMC9700613 36222872

[CR86] Scott, Joan W. 1991. The Evidence of Experience. *Critical Inquiry* 17 (4): 773–797.

[CR87] Seifert, Edith 1985. Kastration und Verneinung. *Die Schwarze Botin*. *Frauenhefte *(27): 25–29.

[CR88] Stadler, Max, Nils Güttler, Niki Rhyner, Mathias Grote, Fabian Grütter, Tobias Scheidegger, Martina Schlünder, Anna M. Schmidt, Susanne Schmidt, Alexander von Schwerin, Monika Wulz und Nadine Zberg (Hg.) 2020. *Gegen|Wissen. Wissensformen an der Schnittstelle von Universität und Gesellschaft*. Zürich: intercom.

[CR89] Stepken, Regine 1980. Ich war krank vor Wut. *Courage. Sonderheft* 2 (2): 12–13.

[CR90] Stöckle, Tina 1983. *Die Irren-Offensive. Erfahrungen einer Selbsthilfe-Organisation von Psychiatrieopfern*. Frankfurt am Main: extrabuch.

[CR91] Strobl, Ingrid 1988. Zwischen Kürbisbrüsten und Entmannung. Bücher von Frauen für Frauen. In: Kristine von Soden (Hg.). *Der große Unterschied. Die Neue Frauenbewegung und die siebziger Jahre.* Berlin: Elefanten Press: 134–140.

[CR92] Susemichel, Lea 2008. Feministische Bildpolitiken. Die Bildergeschichte der an.schläge. In: dies., Saskya Rudigier und Gabi Horak (Hg.). *Feministische Medien. Öffentlichkeiten jenseits des Malestream*. Königstein (Taunus): Ulrike Helmer: 170–179.

[CR93] Susemichel, Lea, Saskya Rudigier, Gabi Horak (Hg.) 2008. *Feministische Medien. Öffentlichkeiten jenseits des Malestreams*. Königstein (Taunus): Ulrike Helmer.

[CR94] Susy 1980. Chemische Zwangsjacke. *Courage. Sonderheft* 2 (2): 28–30.

[CR95] Venske, Regula 1988. Das 5. Treffen schreibender Frauen und die Folgen. *Schreiben* 11 (32): 26–37.

[CR96] Vukadinović, Vojin Saša (Hg.) 2021. *Die Schwarze Botin. Ästhetik, Kritik, Polemik, Satire. 1976–1980*. 2. Aufl. Göttingen: Wallstein.

[CR97] Weinel, Claudia 1984. Feministische Presse in der Bundesrepublik und West-Berlin. Magistraarbeit, Ludwig-Maximilians-Universität München.

[CR98] Wimmer, Jeffrey 2007. *(Gegen‑)Öffentlichkeit in der Mediengesellschaft: Analyse eines medialen Spannungsverhältnisses*. Wiesbaden: VS Verlag für Sozialwissenschaften.

[CR102] *Wissenschaft und Zärtlichkeit* 1981 (10/11).

[CR100] Zöfel, Adelheid 1979. Sylvia Plath. Briefe der besten aller Töchter. *Courage. aktuelle frauenzeitung* 4 (9): 37–41.

[CR99] Z[urmühlen], Sabine 1978. Ein Klaps auf den Rücken. Dorothea K. *Courage. aktuelle frauenzeitung* 3 (3): 5–6.

